# Proteomic and metabolomic profiling of aged pork loin chops reveals molecular phenotypes linked to pork tenderness

**DOI:** 10.1093/jas/skae355

**Published:** 2024-11-20

**Authors:** Logan G Johnson, Chaoyu Zhai, Kenneth J Prusa, Mahesh N Nair, Jessica E Prenni, Jacqueline M Chaparro, Elisabeth Huff-Lonergan, Steven M Lonergan

**Affiliations:** Department of Animal Science, Iowa State University, Ames, IA 50011, USA; Department of Animal Science, University of Connecticut, Storrs, CT 06269-4040, USA; Department of Food Science and Human Nutrition, Iowa State University, Ames, IA 50011, USA; Department of Animal Sciences, Colorado State University, Fort Collins, CO 80523, USA; Department of Horticulture and Landscape Architecture, Colorado State University, Fort Collins, CO 80523, USA; Department of Horticulture and Landscape Architecture, Colorado State University, Fort Collins, CO 80523, USA; Department of Animal Science, Iowa State University, Ames, IA 50011, USA; Department of Animal Science, Iowa State University, Ames, IA 50011, USA

**Keywords:** liquid chromatography-mass spectrometry, metabolomics, pork, proteomics, tenderness

## Abstract

The ability to predict fresh pork tenderness and quality is hindered by an incomplete understanding of molecular factors that influence these complex traits. It is hypothesized that a comprehensive description of the metabolomic and proteomic phenotypes associated with variation in pork tenderness and quality will enhance the understanding and inform the development of rapid and nondestructive methods to measure pork quality. The objective of this investigation was to examine the proteomic and metabolomic profiles of ~2-wk aged pork chops categorized across instrumental tenderness groups. One hundred pork loin chops from a larger sample (*N* = 120) were assigned to one of the four categories (*n* = 25) based on instrumental star probe value (Category A, $\bar{x}=4.23\;kg$, 3.43–4.55 kg; Category B, $\bar{x}=4.79\;kg$, 4.66–5.00 kg; Category C, $\bar{x}=5.43\;kg$, 5.20–5.64 kg; and Category D, $\bar{x}=6.21\;kg$, 5.70–7.41 kg). Soluble protein from ~2 wk aged pork loin was prepared using a low-ionic-strength buffer. Proteins were digested with trypsin, labeled with 11-plex isobaric tandem mass tag reagents, and identified and quantified using a Q-Exactive Mass Spectrometer. Metabolites were extracted in 80% methanol from lyophilized and homogenized tissue samples. Derivatized metabolites were identified and quantified using gas chromatography-mass spectrometry. Between Categories A and D, 84 proteins and 22 metabolites were differentially abundant (adjusted *P* < 0.05). Fewer differences were detected in comparison between categories with less divergent tenderness measures. The molecular phenotype of the more tender (Category A) aged chops is consistent with a slower and less extended pH decline and markedly less abundance of glycolytic metabolites. The presence and greater abundance of proteins in the low-ionic-strength extract, including desmin, filamin C, calsequestrin, and fumarate hydratase, indicates a greater disruption of sarcoplasmic reticulum and mitochondrial membranes and the degradation and release of structural proteins from the continuous connections of myofibrils and the sarcolemma.

## Teaser

Consistently producing high-quality pork remains a significant challenge. However, advancements in proteomic and metabolomic techniques offer new opportunities to better understand and refine the molecular phenotype, enhancing our ability to predict and improve pork quality.

## Introduction

Classification of fresh pork loin based on predicted consumer appeal and value presents opportunities for processors and producers to differentiate their products in value-based marketing. Accurate classification benefits purveyors, chefs, retailers, and consumers while aligning purchases with expectations (Lusk et al., 2018). Pork producers emphasizing high-quality products may gain premiums by meeting fresh pork demand in domestic and export markets in a consistent manner. Measuring, predicting, and controlling pork loin quality depends on understanding pork quality development. The process begins with the live animal and is influenced by early and late postmortem events within muscle and meat. Muscle metabolism directly impacts the changes in the conversion of muscle to meat and contributes to variations observed in the development of meat tenderness. Differences in the abundance of enzymes and metabolites highlight diverse events in the perimortem, early postmortem, and late postmortem muscle, offering insights into molecular phenotypes that affect pork quality. The molecular phenotype of meat promises to be a valuable tool in understanding and predicting pork quality. Therefore, new methods to determine the molecular phenotype can create opportunities to refine and gain knowledge and improve prediction (López-Pedrouso et al., 2020).

Antemortem factors in living muscle metabolism set the stage for early postmortem metabolism (Kastenschmidt et al., 1966). A number of cellular states within living muscle tissue that are drivers of early postmortem metabolism include energy substrate availability, oxidative environments, and changing ionic strength (Yu et al., 2018; Scheffler, 2024). During the conversion of muscle to meat, key enzymes are involved in converting energy stored as glycogen to generate adenosine triphosphate (ATP) through glycogenolysis and glycolysis. The activity of glycogen phosphorylase and glycogen debranching enzyme is necessary to generate the substrate for glycolysis. Phosphofructokinase catalyzes the phosphorylation of fructose-6-phosphate (F6P) from fructose-1,6-diphosphate, a rate-limiting step in glycolysis. England et al. (2014) have suggested that phosphofructokinase activity can directly impact the rate and even extent of pH decline. The phosphagen system is critical in early postmortem muscle energy metabolism and signaling (Scheffler et al., 2014). Creatine kinase can generate ATP quickly from creatine phosphate and adenosine diphosphate (ADP) to stabilize ATP levels for moments early postmortem and perhaps temporarily slow glycolysis. Adenylate kinase can also catalyze the production of ATP using two molecules of ADP to generate ATP and adenosine monophosphate (AMP). Rising the levels of AMP can activate more rapid glycolysis. AMP deaminase can clear AMP to reverse this effect (England et al., 2015).

Rapid and extended pH decline due to glycolysis can negatively impact meat quality (Barbut et al., 2008; Scheffler and Gerrard, 2007). This is likely due to the denaturation of myofibrillar proteins that bind to water, proteinases that degrade structural proteins, and pigments that contribute to fresh meat color. Most recently, the low-ionic-strength soluble protein fraction of pork chops with lower pH and higher purge loss has been shown to have less AMP deaminase than chops with higher pH and less purge (Johnson et al., 2023a). Furthermore, Johnson et al. (2023a) showed a greater abundance of peptides of structural proteins in the chops with less purge loss. The rationale for using this low-ionic-strength soluble protein fraction, where larger intact structural and cytoskeletal proteins are not soluble, is to understand the proteins that are different in abundance based on a meat quality phenotype. This protein fraction could be more readily accessible to higher throughput approaches designed to identify a higher-quality phenotype.

Changes in the myofibrillar, cytoskeletal, and intermediate filament proteins are linked to meat quality. Specifically, proteolysis of these proteins leads to myofibrillar fragmentation and improvement in pork tenderness (Schulte et al., 2019, 2020). Increased desmin proteolysis has been linked to improved water-holding capacity in pork (Kristensen and Purslow, 2001; Melody et al., 2004). Johnson et al. (2023a) reported that the myofibrillar fraction of aged chops with a lower star probe (more tender) contained less desmin, obscurin, titin, and nebulin than the myofibrillar fraction of aged chops with a higher star probe value (less tender). Therefore, it is known that tenderization of pork by postmortem aging is accompanied by degradation of key structural proteins—what is not known is which of these proteins is most important for determining tenderness. A lack of this knowledge hinders the ability to control or, just as importantly, predict tenderness. Defining the proteins and metabolites that contribute to the molecular phenotype associated with the pork quality phenotype will generate answers regarding understanding and predicting meat quality (Huff-Lonergan and Lonergan, 2023). The current study aimed to continue this investigation into defining the molecular phenotype of pork quality by examining the proteomic and metabolomic profile of aged pork chops categorized across instrumental tenderness categories.

## Materials and Methods

The present study was conducted using pork loins collected from a commercial pork harvest facility following standard humane slaughter practices according to United States Department of Agriculture (USDA) guidelines; therefore, Institutional Animal Care and Use approval was not sought.

Pork loins (*N* = 120) were collected at a commercial harvest facility using a deep chilling system at 1 d postmortem on three separate collection days over 3 wk. The pH measurements were taken in the processing plant at the center portion of the loin using a Hanna HI 9025 pH meter (Hanna Instruments, Woonsocket, RI, USA) prior to vacuum packaging in Cryovac bags (Sealed Air, Charlotte, NC, USA) at ~2 Torr vacuum. After aging for 12–14 d at 4 °C, the loins were removed from the packaging and weighed, and the percent purge was weighed and calculated. As previously described, aging time varied from 12 to 14 d to avoid freezing samples before sensory analysis (Carlson et al., 2017a; Johnson et al., 2023a). The pH of the aged samples was measured using a Hanna HI 9025 pH meter (Hanna Instruments) at the center portions of the loin (three individual measurements). Loins were cut into 2.54 cm chops, and two chops were individually vacuum packaged and stored at 4 °C in the dark for 1 d to simulate retail storage. After 1 d, chops and purge were removed from the packaging, and the percent purge was calculated.

Before assigning color scores, chops were allowed to bloom for 20 min. Visual color scores were applied using a 6-point scale (1 = pale pinkish gray/white; 6 = dark purplish red; NPPC, 2000), and visual marbling scores were applied using a 10-point scale (1 = 1% intramuscular fat; 10 = 10% intramuscular fat; NPPC, 2000) to chops from the 10th to 12th rib section by trained evaluators using reference cards as a standard. Hunter L* was measured on one chop from the 10th to 12th rib section of each loin using a Minolta Chroma Meter with a D65 light source, 50-mm aperture, and 2° observer (CR-410; Konica Minolta Sensing Americas Inc., Ramsey, NJ, USA; King et al., 2023).

Two chops were cooked to an internal temperature of 68 °C (Ninja Foodi Grill; AG302, SharkNinja Operating LLC, Needham, MA, USA) to determine chop cook loss and prepare for sensory evaluation. Cubes (1.5 cm^3^) were cut and delivered immediately to each panelist for sensory evaluation. A trained panel (*n* = 4; Institutional Review Board identification number: 14-553-00) evaluated tenderness, chewiness, juiciness, pork flavor, and off-flavor using a 10-point category scale (Carlson et al., 2017b). Lower numbers indicated a lower degree of the given attribute, while higher numbers indicated a higher degree of the given attribute in a sample. An Instron (Instron Industrial Products, Grove City, PA, USA) with a 5-point star probe attachment was used for instrumental tenderness measurements, where three compressions were made across each chop and averaged for a final star probe value (Schulte et al., 2019).

Moisture content was determined using the CEM SMART 6 system (Official Method 2008.06, AOAC International, 2019), and fat content was determined using the CEM ORACLE system (Official Method 2008.06, AOAC International, 2019) (CEM Corporation, Matthews, NC, USA). All analyses were conducted in triplicate and averaged.

### Selection of samples for metabolomic and proteomic analysis

The summary of pork quality from the original 120 pork loins is provided in Supplementary Table 1. From these 120 loins, 100 samples were selected for detailed molecular phenotyping based on 4-star probe categories (*n* = 25 per category; A: most tender 20th percentile of the sample based star probe; B: 25–45th percentile; C: 55–75th percentile; D: least tender 80–100th percentile). The star probe means and ranges for each category (A: $\bar{x}=4.23\;kg$, 3.43–4.55 kg, B: $\bar{x}\;=4.79\;kg$, 4.66–5.00 kg, C: $\bar{x}=5.43\;kg$_,_ 5.20–5.64 kg, and D: $\bar{x}\;=6.21\;kg$, 5.70–7.41 kg) represent a fairly typical range of star probe values in pork (Carlson et al., 2017a, [Bibr CIT0008][Bibr CIT0008]; Schulte et al., 2019). The pork quality phenotype of chops from these categories was reported by Johnson et al. (2023b), and a summary of those data is provided (Supplementary Table 2). The subset of pork chops used (*n* = 100) was from a relatively equal distribution of barrows (*n* = 53) and gilts (*n* = 47) from three sire lines (line 1, *n* = 34, line 2, *n* = 33, and line 3, *n* = 33) collected across three slaughter dates (date 1, *n* = 32, date 2, *n* = 36, and date 3, *n* = 32).

### Protein extraction and preparation

Soluble protein from aged pork loin was prepared using an ice-cold low-ionic-strength buffer (50 mM Tris–HCl [pH 8.5] and 1 mM ethylenediaminetetraacetic acid [EDTA]) as described by Johnson et al. (2023a). Powdered muscle tissue was homogenized in the low-ionic-strength buffer, clarified by centrifugation, filtered through cheesecloth, and only the supernatant was retained. After a Bradford protein assay, all samples were adjusted to 10 mg/mL with ice-cold low-ionic-strength buffer (50 mM Tris–HCl [pH 8.5] and 1 mM EDTA), vortexed, and stored at −80 °C until preparation for liquid chromatography-tandem mass spectrometry (**LC-MS/MS**) analysis.

A separate aliquot from the extract of each sample was adjusted to 6.4 mg/mL with the low-ionic-strength buffer and further diluted to 4 mg/mL with 0.5 vol of protein denaturing buffer (3 mM EDTA, 3% [wt/vol] sodium dodecyl sulfate [**SDS**], 30% [vol/vol] glycerol, 0.001% [wt/vol] pyronin Y, and 30 mM Tris–HCl [pH 8.0]) and 0.1 vol of 2-mercaptoethanol (Huff-Lonergan et al., 1996a, 1996b). Samples were vortexed, heated on a dry heat block for 15 min at ~50 °C, and stored at −80 °C. Confirmation of equal protein concentrations between samples was achieved using 15% sodium dodecyl sulfate-polyacrylamide gel electrophoresis (**SDS-PAGE**) with a 5% stacking gel, as described by Johnson et al. (2023a).

### Western blotting

The low-ionic-strength buffer extracts in protein denaturing buffer (4 mg/mL) from Category A (*n* = 25) and Category D (*n* = 25) were used to evaluate desmin with western blotting using one-dimensional SDS-PAGE (15% acrylamide with a 5% acrylamide stacking gel). A pooled reference, with equal proportions of extracts from Categories A (*n* = 5) and D (*n* = 5), was prepared and included on each gel. The remaining lanes were loaded with 40 µg of protein per sample. Samples were randomly assigned to gels.

Protein fractionation, blotting, and detection using western blotting were conducted as described by Johnson et al. (2023a). Briefly, proteins were resolved at 130 V for ~360 V/h in Hoefer 260 Mighty Small II units (Hoefer, Inc., Holliston, MA, USA). Following electrophoresis, proteins were transferred to polyvinylidene difluoride (**PVDF**) membranes (0.2-μm pore size) using TE-22 Mighty Small Transphor units (Hoefer, Inc., Bridgewater, MA, USA) as described by Johnson et al. (2023a). Post transfer, membranes were blocked for 1 h in PBS-Tween (80 mM Na_2_HPO_4_, 20 mM NaH_2_PO_4_, 100 mM NaCl, and 0.1% [vol/vol] polyoxyethylene sorbitan monolaurate [Tween 20]) containing 5% (wt/vol) nonfat dry milk. Incubation with a polyclonal rabbit anti-desmin antibody produced at Iowa State University (Huff-Lonergan et al., 1996b) diluted 1:40,000 in PBS-Tween buffer was conducted at 4 °C for ~18 h. After washing in PBS-Tween, the secondary goat anti-rabbit-HRP antibody (31460; Thermo Scientific, Waltham, MA, USA) was diluted 1:15,000 in PBS-Tween buffer, and membranes were incubated with the secondary antibody for 1 h at room temperature. After washing in PBS-Tween, immunoreactive desmin protein bands were detected using a chemiluminescent detection kit (ECL Prime; GE Healthcare, Piscataway, NJ, USA), and images of blots were obtained and analyzed using a ChemiImager 5500 (Alpha Innotech, San Leandro, CA, USA) and Alpha Ease FC software (version 3.03; Alpha Innotech). Densitometry was used to quantify the intact 55-kDa desmin band, and comparisons were made by taking the ratio of the measured protein band to the internal reference. At least two technical replicates of each sample (*n *= 50) were conducted on separate days. A western blot of companion myofibrillar (prepared as described in Johnson et al. 2023b) and low-ionic-strength protein extracts from the same pork chop was prepared as described above.

### Tandem mass tag analysis

The soluble protein extracts were processed for Tandem Mass Tag (**TMT**) LC-MS/MS analysis as described by Johnson et al. (2023a). A Master Control sample was generated where 5 µg of each sample was pooled, reduced, alkylated, and digested with trypsin according to the manufacturer’s directions. The Master Control sample was desalted using a C18 MicroSpin Column (SEM SS18V, Nest Group, Inc, Ipswich, MA, USA) and dried. The Master Control was reconstituted in 100 mM triethyl ammonium bicarbonate (**TEAB**), and the concentration of the peptides was determined using a Pierce Colorimetric kit (Thermo Scientific). The TMT11-131C reagent (A37724, Thermo Scientific) was reconstituted, and 100 µg of the Master Control was incubated with 0.8 mg of the TMT11-131C reagent for 1 h. The reaction was quenched by adding 8 µL of 5% hydroxylamine in 100 mM TEAB for 15 min.

Each sample (25 µg) was reduced, alkylated, and digested with trypsin according to the manufacturer’s directions. Trypsin digestion was quenched with formic acid, and samples were dried and subsequently reconstituted in 40 µL of 5% acetonitrile and 0.1% trifluoroacetic acid and desalted using MicroSpin Columns with C18 silica (SEM SS18V, Nest Group, Inc.). The samples were dried and reconstituted in 100 µL of 100 mM TEAB. The peptide concentration was quantified using a Pierce Colorimetric kit (Thermo Scientific). Each sample (25 µg) was incubated with 0.2 mg of a TMT10plex Label Reagents (90110, Thermo Scientific) for 1 h. The reaction was quenched by adding 8 µL of 5% hydroxylamine in 100 mM TEAB for 15 min. The samples (*N* = 100) were randomly assigned to 10 different runs, with 10 samples per run, and star probe categories were manually balanced across each run. Within a run, samples were randomly assigned to one of the TMT10plex Label Reagents (90110, Thermo Scientific). The digested and labeled peptides from each sample (3 µg) within a run and the Master Control (3 µg) were pooled (33 µg total) and dried. The pooled runs were reconstituted with 33 µL of 5% acetonitrile and 0.1% trifluoroacetic acid.

Chromatographic separation of peptides was achieved using an EASY-nLC 1200 (Thermo Scientific) system with an integrated autosampler, as described in Johnson et al. (2023a). The elution gradient consisted of a linear gradient of 0% to 35% Buffer B (0.1% formic acid in acetonitrile) over 240 min, a linear gradient of 35% to 70% Buffer B over 20 min, and a linear gradient of 70% to 100% Buffer B over 4 min at a flow rate of 300 nL/min. Peptides were detected using a Q-Exactive Hybrid Quadrupole-Orbitrap mass spectrometer equipped with a Higher Energy Collisional Dissociation (**HCD**) cell and a Nanospray Flex Ion source. Data were acquired with an automatic gain control (**AGC**) target of 1 × 10^6^ and a maximum injection time of 80 ms. The MS1 scans were acquired at 70,000 resolving power, and precursor ions were selected within a scan range of 400–2,000 *m/z* during positive ionization mode. The MS2 scans were collected with an isolation window of 1.2 *m*/*z* and fragmented at a 32% normalized collision energy. The ions were analyzed at 35,000 resolving power with an AGC target of 1 × 10^5^ and a maximum injection time of 50 ms.

### Proteomic data processing

The data were analyzed using Proteome Discoverer (version 2.5.0.400; Thermo Scientific). Spectra files were searched using Sequest HT and Mascot against a *Sus scrofa* UniProt database. Search parameters of peptide datasets included a 10 ppm precursor mass tolerance and a 0.02 Da fragment mass tolerance for b and y ions produced by HCD fragmentation. Data were searched with the static carbamidomethyl modification of cysteine residues, static TMT-6plex modifications of peptide N-termini and lysine residues, dynamic methionine oxidation, and dynamic deamination of asparagine and glutamine residues. Identified peptides were filtered to a 1% false discovery rate (**FDR**). The peptides were then matched to proteins and were filtered to a 1% FDR on the protein level. Proteins were grouped using the maximum parsimony principle from all retained peptide-spectrum matches (PSM) using the default settings of Proteome Discoverer, as described previously (Johnson et al., 2023a). The FASTA sequence of identified proteins in the experiment labeled as “Uncharacterized Protein” was searched in UniProt BLAST (The UniProt Consortium, 2023) with default parameters. The protein with the lowest expectation value (E) threshold was used to rename the identified protein in the experiment.

### Preparation for metabolite analysis

Individual samples from each loin chop (~150 g) were homogenized in liquid nitrogen and then freeze-dried. Freeze-dried samples (27 mg) were extracted with 1 mL of high-performance liquid chromotography (HPLC) grade 80% methanol (**MeOH**) in water. Samples were vortexed on a plate shaker at 4 °C for 2 h, held at −80 °C for 2 h, and clarified via centrifugation (2,500 × *g*). A portion of the supernatant (100 μL) was dried under nitrogen and resuspended in 100 µL of 4:4:1 MeOH:acetonitrile (**ACN**):water. Samples were incubated overnight at −20 °C and centrifuged at 12,700 × *g* for 15 min at 4 °C. A pooled quality control (**QC**) solution was created by transferring 10 μL of each sample into a separate glass vial. A portion of the supernatant (30 µL) of each sample was transferred to a new vial and dried under nitrogen. Samples were stored at −80 °C until derivatization.

For derivatization, the dried samples were resuspended in 50 µL of pyridine containing 25 mg/mL of methoxyamine hydrochloride (Sigma-Aldrich, St. Louis, MO, USA) incubated at 60 °C for 1 hr, sonicated for 10 min, and incubated for an additional 1 hr at 60 °C. Incubation continued for 45 min after adding 50 µL of MSTFA + 1% TMCS (Thermo Scientific). Each sample was briefly centrifuged, cooled to room temperature, and 80 µL of the supernatant was transferred to a 150 µL glass insert in an autosampler vial.

### Data acquisition for nontargeted analysis

One microliter of the derivatized sample was injected at a 1:12 split ratio and 1.0 mL/min helium gas flow. A Clarus 690 gas chromatography system (PerkinElmer, Waltham, MA, USA) coupled to a PerkinElmer Clarus SQ 8T mass detector was used to detect small semi and nonvolatile molecules. Separation was conducted with a TG-5MS column (Thermo Scientific, 30 m × 0.25 mm × 0.25 μm). The oven profile consisted of an 80 °C hold for 30 s, ramping 15 °C/min to 330 °C, with an 8-min hold at the end of the run. Masses between 50 and 620 *m/z* were scanned at 4 scans/s after electron impact ionization operating at 70 eV. The injector temperature was held at 285 °C, the transfer line was set to 300 °C, and the source was set to 260 °C. A pooled QC was run for every 6th sample to assess and control analytical variation.

### Metabolomic data processing

Gas chromatography-mass spectrometry data files were converted to .cdf format and processed by XCMS in R (Smith et al., 2006; Tautenhahn et al., 2008; Mahieu et al., 2016). Normalization was conducted on all samples based on run order vs. QC feature intensities to account for instrumental drift followed by the total ion current (**TIC**). Peak deconvolution into spectral clusters occurred in RAMClustR to facilitate metabolite annotation (Broeckling et al., 2014). Metabolites were annotated in RAMSearch (Broeckling et al., 2016) based on matching of the retention index and mass spectra data with external databases, including Golm Metabolome database (Hummel et al., 2007, 2013) and NISTv17 (http://www.nist.gov) (Broeckling et al., 2016).

### Statistical analysis

All statistical analyses were performed using R Statistical Software (v.4.2.0, R Core Team, 2023). Within the proteomic data, the reporter ion intensities were normalized to the total ion count within each run. The ratio of each TMT10-plex reporter ion intensities relative to the TMT11-131C Master control within a run for an identified protein was calculated. In subsequent analysis, only proteins identified in at least half of the samples (*n* > 49) containing at least two unique peptides were included. The ratios were log_2_ transformed and normalized to the median value within a sample. Moderated *t*-tests were used to make pairwise comparisons between star probe categories using the limma package (Ritchie et al., 2015). The Benjamini–Hochberg multiple testing adjustment was used to control the FDR and control for multiple testing (Benjamini and Hochberg, 1995). Proteins were considered differentially abundant at an adjusted *P *< 0.05, and trends were denoted by 0.05 ≤ *P* < 0.10.

Normalization of metabolomic data was first conducted on all samples based on run order vs. QC feature intensities to account for instrumental drift followed by the TIC. The data were further log_2_ transformed and normalized to the median value within a sample prior to statistical analysis. Moderated *t*-tests were used for pairwise comparisons between star probe categories using the limma package (Ritchie et al., 2015). The Benjamini–Hochberg multiple testing adjustment was used to control the FDR and control for multiple testing (Benjamini and Hochberg, 1995). Metabolites were considered differentially abundant at an adjusted *P *< 0.05, and trends were denoted by 0.05 ≤ *P* < 0.10.

Summary statistics of each pork quality and sensory attribute were calculated and reported in Supplementary Table 1. A one-way analysis of variance was used with the fixed effect of star probe category (*n* = 25/category) on pork quality and sensory attributes as well as desmin immunoreactive bands in samples from Category A and D chops. Estimated marginal means were computed using the emmeans function to make pairwise comparisons between star probe categories. The fixed effects of sex, harvest date, and sireline were tested separately for each attribute and were reported in Supplementary Table 2. Significance was denoted by a *P *< 0.05, and trends were denoted by 0.05 ≤ *P* < 0.10.

## Results

**Figure 1.**
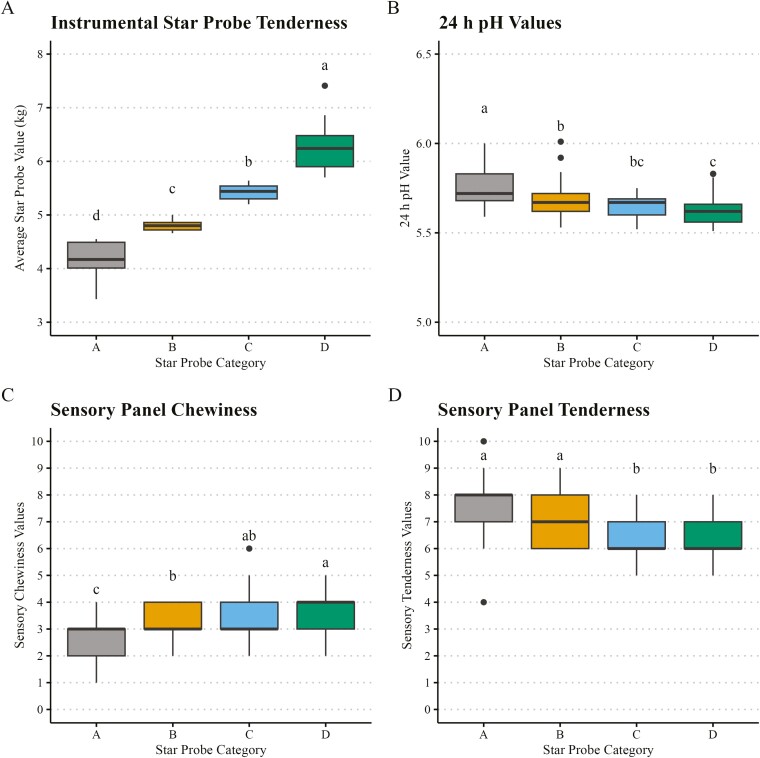
Box and whisker plots that summarize ~2-wk postmortem pork loin meat quality and sensory traits across loins in each star probe category (*n* = 25 per category). (A) Instrumental star probe tenderness measured on cooked chops with a 5-pointed Instron probe (Carlson et al., 2017a), (B) pH of pork loins measured at 24 h postmortem and trained sensory panel scores of (C) sensory chewiness, and (D) sensory tenderness based on a 10-point line scale. Star probe Categories A, B, C, and D were classified by instrumental star probe tenderness. Category A: $\bar{x}=4.23\;kg$, 3.43–4.55 kg; Category B: $\bar{x}\;=4.79\;kg$, 4.66–5.00 kg; Category C: $\bar{x}=5.43\;kg$, 5.20–5.64 kg; Category D: $\bar{x}\;=6.21\;kg$, 5.70–7.41 kg. Means with different lowercase alphabets (a–d) are significantly different (*P* < 0.05).

**Figure 2.**
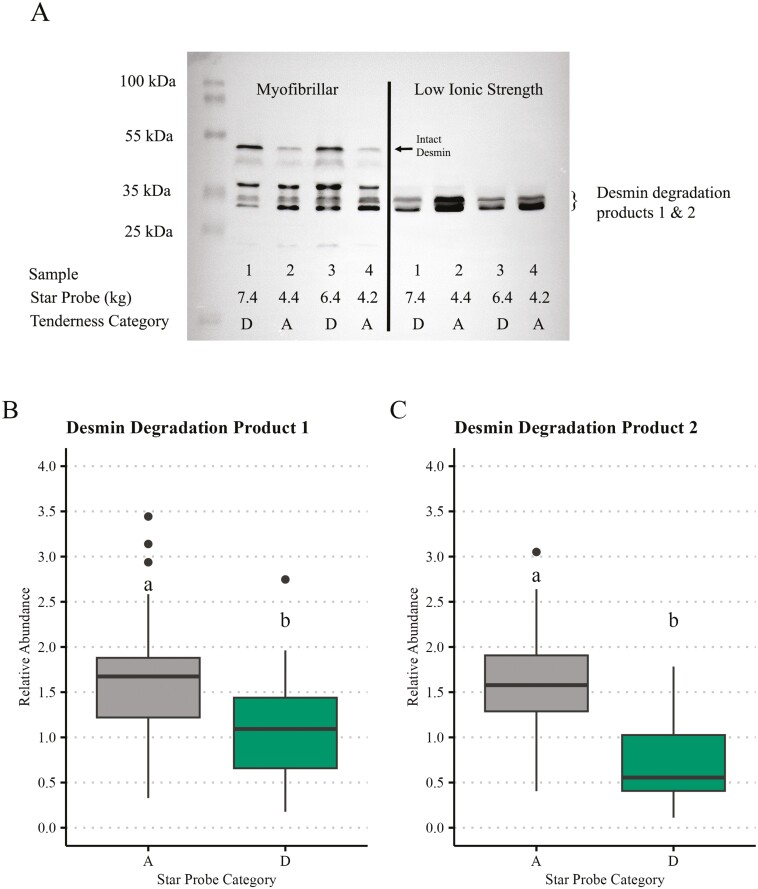
(A) Western blot of desmin from the myofibrillar (lanes 2–5) and low-ionic-strength soluble (lanes 6–9) protein fractions of aged pork loins in Categories A and D. The profile of desmin between the myofibrillar and low-ionic-strength fraction is distinct, yet related as samples 1, 2, 3, and 4 are the same samples across these two protein fractions. No intact desmin was detected in the low-ionic-strength soluble fraction, but immunoreactive bands are labeled desmin degradation products 1 and 2. (B) and (C) Box and whisker plots of the relative abundance of desmin degradation product 1 and 2 in the low-ionic-strength extracts from chops in Category A and D (*n* = 25 per category), respectively. Means with different lowercase alphabets (a and b) are significantly different (*P* < 0.05).

The current study is one of the most extensive individual studies, regarding the number of observations, combining pork’s metabolomic and proteomic phenotypes associated with pork tenderness. The summaries of the pork quality, composition, and sensory phenotypes in the original 120 pork loins (Supplementary Table 1) and the means across the star probe categories (*n* = 100; Supplementary Table 2) illustrated in Figure 1 demonstrate the diversity of the original samples and the phenotypic differences across the quality categories. True to the star probe category assignment, chops in each category differed (*P* < 0.01), with Category A chops demonstrating the lowest star probe and chops in Category D demonstrating the highest star probe values. Chops in Category A had the highest pH (*P* < 0.01) and were less chewy (*P* < 0.01) than chops in other categories. Based on sensory tenderness scores, chops in Categories A and B were more tender (*P* < 0.01) than chops in Categories C and B. Chops in Category A had greater lipid content and higher marbling scores (*P* < 0.05) than chops in all other categories. In the myofibrillar fraction of the aged chops from a prior study, intact desmin was more abundant (*P* < 0.01) in Category D than in Category A chops (Supplementary Table 1; Johnson et al., 2023b). Intact desmin is easily detected in myofibrillar protein extracts (Figure 2A), but no intact desmin is present in the low-ionic-strength extracts. Two desmin degradation fragments (designated desmin degradation products 1 and 2) detected soluble in the low-ionic-strength buffer were more abundant (*P* < 0.01) in the Category A chops than D chops (Figure 2B and Figure 2C).

The identified proteins had to be present in more than half of the samples and have at least two unique peptides to be retained in the study. Four hundred thirty-nine proteins (based on accession number) were included in the analysis. Forty-six metabolites were included in the analysis. Notably, the most differentially abundant proteins and metabolites were detected in the categories with the most extreme quality phenotypes (Category A vs. D). Eighty-four proteins and twenty-two metabolites were different (adjusted *P* < 0.05) in comparing Categories A and D (Table 1), illustrating that categorization based on star probe phenotype resulted in a greater difference in the molecular phenotype of the aged samples.

**Table 1.** Summary of significantly different metabolites and proteins in comparison of star probe categories^1^

Comparison	Number of differentially abundant metabolites^2^	Number of differentially abundant proteins^3^
A vs. B	0	9
A vs. C	12	33
A vs. D	22	84
B vs. C	0	0
B vs. D	0	6
C vs. D	0	0

^1^Categories A, B, C, and D were classified by instrumental star probe tenderness. Category A (*n* = 25): $\bar{x}=4.23\;kg$, 3.43–4.55 kg; Category B (*n* = 25): $\bar{x}\;=4.79\;kg$, 4.66–5.00 kg; Category C (*n* = 24): $\bar{x}=5.43\;kg$, 5.20–5.64 kg; Category D (*n* = 25): $\bar{x}\;=6.21\;kg$, 5.70–7.41 kg.

^2^Number of metabolites included in the analysis (*n* = 46).

^3^Number of proteins included in the analysis (*n* = 307).

All metabolite identifications were considered to be either level 2 (putative) based on matching fragmentation spectra against spectral libraries or level 3 (chemical class) based on partial matching against spectral libraries. Of the 46 metabolites detected, 22 were significantly different (adjusted *P* < 0.05) in Category A vs. Category D (Table 2), with a notable result of a greater abundance of sugars in Category D. Thirteen metabolites, including amino acids and glutamic acids, were more abundant (adjusted *P* < 0.05) in Category A (Table 2). Fewer differences were noted in the comparison of Category A vs. Category C, yet Category C had a greater abundance (adjusted *P* < 0.05) of sugars, including mannose-6-phosphate, glucose-6 phosphate, fructose, and sorbose. Molecules detected, but not different in comparing Categories A and D, are summarized in Supplementary Table 3.

**Table 2.** Summary of the significantly different metabolites between Categories A and C and Categories A and D^1^^,^^2^

	Log_2_ fold difference		Adjusted *P*-values	
	A vs. C	A vs. D	A vs. C	A vs. D
More in Category A than D				
Tryptophan	0.54	0.59	**<0.01**	**<0.01**
Serine	0.11	0.44	0.38	**<0.01**
Leucine	0.18	0.41	0.22	**<0.01**
Valine	0.17	0.36	0.14	**<0.01**
Threonine	0.11	0.36	0.33	**<0.01**
Tyrosine	0.39	0.35	**<0.01**	**<0.01**
Lysine	0.26	0.33	**0.03**	**<0.01**
Phenylalanine	0.31	0.32	**<0.01**	**<0.01**
Glutamic acid	0.24	0.32	0.08	**<0.01**
Isoleucine	0.22	0.31	**<0.05**	**<0.01**
Methionine	0.34	0.31	**<0.01**	**<0.01**
Ornathine	0.26	0.29	0.08	**0.02**
Hypoxanthine	0.24	0.26	0.08	**0.04**
More in Category D than A				
Mannose-6-phosphate	−1.22	−1.64	**<0.01**	**<0.01**
Glucose-6-phosphate	−1.08	−1.58	**<0.05**	**<0.01**
Glucosamine-6-phosphate	−1.06	−1.55	**0.01**	**<0.01**
Fructose	−0.68	−1.09	**0.02**	**<0.01**
Talose	−0.66	−1.07	**0.03**	**<0.01**
Sorbose	−0.61	−0.98	**<0.01**	**<0.01**
Cholesterol	−0.27	−0.72	0.24	**<0.01**
Galactose	−0.27	−0.63	0.12	**<0.01**
Glucose	−0.13	−0.44	0.33	**<0.01**
Lactic acid	−0.01	−0.22	0.91	0.051

^1^Categories A, B, C, and D were classified by instrumental star probe tenderness. Category A (*n* = 25): $\bar{x}=4.23\;kg$, 3.43–4.55 kg; Category B (*n* = 25): $\bar{x}\;=4.79\;kg$, 4.66–5.00 kg; Category C (*n* = 24): $\bar{x}=5.43\;kg$, 5.20–5.64 kg; Category D (*n* = 25): $\bar{x}\;=6.21\;kg$, 5.70–7.41 kg.

^2^Metabolite profile of Category B did not differ from any other category. Bold values indicate significant differences between Categories (Adjusted *P*-value < 0.05).

In comparing the two most tender categories, eight proteins were more abundant (adjusted *P* < 0.05) in Category A than Category B chops, whereas only β-enolase was more abundant (adjusted *P* < 0.05) in Category B chops than Category A chops (Table 3). A greater magnitude of difference in star probe was associated with an increasing number of significantly different proteins and a greater magnitude of difference in protein abundance, demonstrating a link between the pork quality phenotype and the proteomic profile. For example, the log_2_-fold difference in filamin C was 0.299, 0.429, and 0.590 in A vs. B, A vs. C, and A vs. D comparisons (adjusted *P* < 0.05; Table 3), thus drawing a connection between pork quality and proteomic phenotypes. In comparing Categories A and D, structural, contractile, sarcoplasmic reticulum, chaperone, and metabolic proteins were differentially abundant in the low-ionic-strength fraction (Table 4). Proteins detected, but not different in comparing Categories A and D, are listed in Supplementary Table 4.

**Table 3.** Summary of significant (*P* < 0.05) proteomic differences, where log_2_-fold differences are reported for proteins that were different across more than one comparison^1^

Description	Accession	Log_2_ fold difference			
		A vs. B	A vs. C	A vs. D	B vs. D
2-Phospho-d-glycerate hydrolyase (**β-enolase**)	A0A4X1USV7	−0.174	−0.232	−0.275	
Titin isoform X6	A0A480SN35	0.171	0.262	0.333	
Filamin C	F1SMN5	0.299	0.429	0.590	0.291
CCT-θ	A0A480X895	0.333	0.366	0.482	
Heat shock 27 kDa protein	A0A5S6G3Y8	0.415	0.563	0.712	
Succinate-CoA ligase	A0A5G2R4W7	0.461	0.550	0.668	
Uncharacterized protein (**titin**)	A0A5G2QM05	0.510	0.814	1.125	0.615
Troponin I	B3VI70	0.621	0.776	0.934	
(β)-Crystallin	A0A4X1TDZ5	0.624	0.754	0.971	
Titin	A0A480J3J1		−0.772	−1.239	−0.745
Uncharacterized protein (**LIM domain-binding protein 3**)	A0A287A435		−0.255	−0.392	−0.237
Uncharacterized protein (**obscurin**)	A0A5G2QZ79		0.286	0.396	0.269
AMP deaminase	A6NA29		0.639	0.866	0.457
Protein S100	A0A4X1VVF5		−0.343	−0.336	
Myosin light chain kinase 2, skeletal/cardiac muscle	A0A4X1STF9		−0.307	−0.297	
GST class-pi (**glutathione S-transferase**)	A0A287BQ81		−0.222	−0.275	
Triosephosphate isomerase	A0A288CFT0		−0.202	−0.225	
Maillard deglycase	Q0R678		−0.142	−0.236	
Ubiquitin-conjugating enzyme E2 V2	A0A5G2R6G7		−0.138	−0.216	
HATPase_C domain-containing protein	A0A287BDM6		0.247	0.305	
Actin, alpha skeletal muscle	A0A481CYB2		0.253	0.192	
ATP-dependent 6-phosphofructokinase	A0A287B8N1		0.317	0.372	
Isocitrate dehydrogenase (NADP), mitochondrial (Fragment)	P33198		0.377	0.610	
Actinin α 3	A0A5G2RET9		0.463	0.426	
Heat shock protein β-8	A0A286ZID4		0.477	0.646	
Actinin α 2	F1RHL9		0.493	0.512	
Dynamin-type G domain-containing protein (**sarcalumenin**)	A0A4X1VRP4		0.497	0.783	
Fumarate hydratase, mitochondrial	A0A4X1THY9		0.501	0.663	
Troponin C, skeletal muscle	P02587		0.548	0.662	
Desmin	P02540		0.561	0.761	
Uncharacterized protein (**14-3-3 protein gamma**)	F2Z4Z1		0.583	0.604	
SET and MYND domain containing 1	A0A287ALA4		0.723	0.907	
Calsequestrin	F1RJW7		0.903	1.152	

^1^Categories A, B, C, and D were classified by instrumental star probe tenderness. Category A (*n* = 23): $\bar{x}=4.23\;kg$, 3.43–4.55 kg; Category B (*n* = 25): $\bar{x}\;=4.79\;kg$, 4.66–5.00 kg; Category C (*n* = 23): $\bar{x}=5.43\;kg$, 5.20–5.64 kg; Category D (*n* = 24): $\bar{x}\;=6.21\;kg$, 5.70–7.41.

Uncharacterized proteins were named by searching the FASTA sequence in UniProt BLAST (The UniProt Consortium, 2023). Those assigned names are within parentheses and in bold.

**Table 4.** Summary of proteins that were differentially abundant in comparison of the lowest star probe category (Category A) to the highest star probe category (Category D)^1^

Protein description	Accession number	Gene name	Sequence coverage^2^	Unique peptides	Log_2_ fold difference	Adjusted *P*-value
Structural						
Uncharacterized protein (**titin**)	A0A5G2QM05		61	27	1.125	<0.001
Desmin	P02540	DES	52	22	0.761	<0.001
Filamin C	F1SMN5	FLNC	31	56	0.590	<0.001
Actinin α 2	F1RHL9	ACTN2	33	17	0.512	0.002
Actinin α 3	A0A5G2RET9	ACTN3	52	33	0.426	0.024
Uncharacterized protein (**obscurin**)	A0A5G2QZ79	OBSCN	21	10	0.396	<0.001
Titin isoform X6	A0A480SN35		14	249	0.333	<0.001
Vimentin	P02543	VIM	41	15	0.305	0.041
Actin, α skeletal muscle	A0A481CYB2		53	6	0.192	0.040
Plectin isoform 1a	A0A480IPA8		42	4	0.136	0.009
Uncharacterized protein (**Titin**)	A0A4X1U902		8	10	0.129	0.004
Nebulin	F1SHX0	NEB	25	4	−0.263	0.001
Nebulin isoform 3	A0A481BBQ7		24	3	−0.701	0.008
Titin	A0A480J3J1		22	2	−1.239	<0.001
Contractile						
Troponin C, skeletal muscle	P02587	TNNC2	60	9	0.662	<0.001
Troponin I	B3VI70	TnI-F4	34	5	0.934	<0.001
Troponin T, fast skeletal muscle	Q75NG8	tnnt3	22	8	0.450	<0.001
Uncharacterized protein (**myosin regulatory light chain 2**)	A0A4X1TZM9	MYLPF	58	10	0.316	0.001
Calcium transport proteins						
Calcium-transporting ATPase 1	A0A5G2R940	ATP2A1	43	24	0.621	0.002
Calcium-transporting ATPase 2	A0A4X1U5D4	ATP2A2	19	6	0.544	<0.001
Calsequestrin 1	F1RJW7	CASQ1	30	8	1.152	<0.001
Sarcoplasmic reticulum proteins						
Annexin A7	F1SU59	ANXA7	23	10	−0.201	0.003
Dynamin-type G domain-containing protein (**sarcalumenin**)	A0A4X1VRP4	SRL	26	17	0.783	<0.001
Junctophilin-1	F1RWK0	JPH1	17	9	−0.192	0.040
Protein S100	A0A4X1VVF5	S100A6	24	3	−0.336	0.023
Annexin A11	F1S2E2	ANXA11	19	8	−0.307	0.029
Chaperone proteins						
α-Crystallin β chain (**heat shock protein β-5**)	A0A4X1TDZ5	CRYAB	27	4	0.971	<0.001
CCT-θ (**CCT-8**)	A0A480X895		21	9	0.482	<0.001
Galectin	A0A5G2QD67	LGALS1	51	5	0.232	0.037
HATPase_c domain-containing protein (**heat shock protein C-3**)	A0A287BDM6	HSP90AB1	35	9	0.305	<0.001
Heat shock 27 kDa protein (**heat shock protein β-1**)	A0A5S6G3Y8	HSPB1	16	4	0.712	<0.001
Heat shock protein β-8	A0A286ZID4	HSPB8	10	2	0.646	<0.001
T-complex protein 1 subunit η (**CCT-7**)	A0A480KDY2		6	3	0.265	0.007
Metabolic						
ATP synthase F1 subunit δ	A0A4X1VPE5	ATP5F1D	14	2	−0.257	0.022
ATP synthase subunit β	A0A481D232		61	22	−0.200	0.013
Glycerol-3-phosphate dehydrogenase [NAD(+)]	A0A287BDV9	GPD1	84	20	−0.218	<0.001
Medium-chain specific acyl-CoA dehydrogenase, mitochondrial	P41367	ACADM	35	10	−0.297	0.032
Mitochondrial aldehyde dehydrogenase 2	B2ZF47	ALDH2	53	19	−0.185	0.043
Myosin light chain kinase 2, skeletal/cardiac muscle	A0A4X1STF9	MYLK2	22	10	−0.297	0.004
Perilipin 4	A0A287AV63	PLIN4	36	6	0.350	0.002
SH3 domain-binding glutamic acid-rich protein isoform a (Fragment)	A0A480TH91		37	4	−0.298	0.027
AMP metabolism						
Adenylate kinase isoenzyme 1	A0A286ZQ79	AK1	58	18	−0.260	<0.001
Adenylosuccinate lyase	F6QAM9	ADSL	22	8	−0.216	0.044
AMP deaminase	A6NA29		33	22	0.866	<0.001
Glycogen metabolism and glycolysis						
2-Phospho-d-glycerate hydrolyase (**α-enolase)**	A0A4X1W8R1		48	11	−0.378	0.004
2-Phospho-d-glycerate hydrolyase (**β-enolase**)	A0A4X1USV7	ENO3	67	20	−0.275	<0.001
4-α-Glucanotransferase	F1S557	AGL	78	11	−0.116	0.041
ATP-dependent 6-phosphofructokinase	A0A287B8N1	PFKM	47	29	0.372	0.001
Fructose-bisphosphatase	A0A4X1V2J1		52	14	−0.311	0.038
Fructose-bisphosphate aldolase	A0A4X1U0N5	ALDOA	79	3	−0.157	0.002
Fructose-bisphosphate aldolase	A0A4X1TPV9	ALDOC	25	4	−0.251	0.040
l-Lactate dehydrogenase	A0A480X8T8		76	26	−0.233	0.023
Multifunctional fusion protein	A0A287AQJ5	PKM	36	5	−0.276	<0.001
Phosphoglycerate kinase	F1RPH0	PGK1	76	21	−0.264	<0.001
Phosphoglycerate mutase 2	B5KJG2	PGAM2	80	15	−0.249	<0.001
Triosephosphate isomerase	A0A288CFT0	TPI1	80	27	−0.225	0.003
Uncharacterized protein (**phosphoglycerate mutase 1**)	A0A4X1TAN5	PGM1	75	5	−0.101	0.033
Oxidative metabolism						
Fumarate hydratase, mitochondrial	A0A4X1THY9	FH	30	11	0.663	<0.001
Isocitrate dehydrogenase [NADP], mitochondrial (fragment)	P33198	IDH2	36	13	0.610	<0.001
Succinate--CoA ligase [ADP/GDP-forming] subunit α, mitochondrial	A0A5G2R4W7	SUCLG1	7	2	0.668	<0.001
Antioxidants						
Maillard deglycase	Q0R678	PARK7	80	15	−0.236	<0.001
Hemopexin	F1RMN7	HPX	33	2	−0.175	0.014
LIM domain						
Four and a half LIM domains protein 1 isoform 5	F6PXR6		60	16	0.601	<0.001
Uncharacterized protein (**LIM domain-binding protein 3**)	A0A287A435	LDB3	47	15	−0.392	<0.001
Uncategorized						
14-3-3 domain-containing protein (**14_3_3 protein epsilon**)	A0A5G2QSR7	CRK	40	8	0.583	0.001
CXXC motif containing zinc-binding protein	F1S765	CZIB	36	5	−0.197	0.041
GST class-pi (**glutathione *S*-transferase**)	A0A287BQ81	LOC100739508	55	9	−0.275	<0.001
Leukotriene A(4) hydrolase	A0A480LQK0		29	14	0.288	0.003
*S*-Formylglutathione hydrolase	Q9GJT2	ESD	59	11	−0.197	0.024
Uncharacterized protein (**14_3_3 protein gamma**)	F2Z4Z1	YWHAG	54	8	0.604	<0.001
Uncharacterized protein (**MTH 938 domain containing Protein**)	A0A4X1U924	AAMDC	47	6	−0.157	0.040
Uncharacterized protein (**AHNAK nucleoprotein**)	A0A4X1VKL9		26	17	−0.120	0.040
α-2-HS-glycoprotein	A0A4X1UAD2	AHSG	36	9	−0.260	0.041
Geranylgeranyl transferase type-2 subunit α	A0A4X1SQT5	RABGGTA	3	2	0.512	0.043
Macro domain-containing protein	A0A4X1VJE3	MACROD1	36	7	−0.151	0.040
Obg-like ATPase 1	A0A4X1UNV8	OLA1	43	14	0.212	0.008
PDZ domain-containing protein	A0A4X1V8J5	SYNPO2	9	9	−0.326	0.040
Proteasome subunit β	A0A4X1UGB1	PSMB3	16	3	−0.299	0.017
Reticulon	A0A4X1W6J5	RTN4	30	5	0.453	0.019
Serotransferrin	P09571	TF	68	43	−0.279	0.022
SET and MYND domain containing 1	A0A287ALA4	SMYD1	11	3	0.907	0.004
Ubiquitin-conjugating enzyme E2 V2	A0A5G2R6G7	UBE2V2	25	2	−0.216	<0.001
Uncharacterized protein (**protein phosphatase 1 regulatory inhibitor subunit 14C**)	A0A4X1VL95	PPP1R14C	19	2	−0.473	0.039
Uncharacterized protein (**serine/threonine kinase receptor-associated protein**)	I3LEQ7	STRAP	13	4	0.377	0.022

^1^Categories A and D were classified by instrumental star probe tenderness. Category A (*n* = 23): $\bar{x}=4.23\;kg$, 3.43–4.55 kg; Category D (*n* = 24): $\bar{x}\;=6.21\;kg$, 5.70–7.41 kg.

^2^Sequence coverage in percent.

Uncharacterized proteins were named by searching the FASTA sequence in UniProt BLAST (The UniProt Consortium, 2023). Those assigned names are within parentheses and in bold.

## Discussion

The current experiment evaluated proteins soluble in a low-ionic-strength buffer and metabolites soluble in water and methanol. The rationale for emphasizing these fractions is that they are generally accessible and could be used in larger-scale, high-throughput methods to achieve accurate quality evaluation. In understanding and defining these molecular phenotypes, this information will help to deepen the knowledge of biochemical factors that impact pork quality. The potential to use these molecular features in future work to more rapidly and accurately predict pork meat quality demonstrates great potential, given these molecular features likely change and more accurately describe the differences in the actual meat quality phenotype. Numerous aspects of this work have yet to be validated in subsequent populations over different aging times; however, the use of these molecular phenotypes exists to inform genetic improvement and procedures to assign value to fresh pork with nondestructive methods.

### Structural proteins

Many myofibrillar, cytoskeletal, intermediate filament, and costameric proteins were identified in aged pork loin’s low-ionic-strength buffer extract (Tables 3 and 4; Supplementary Table 2). The presence of peptides aligning with structural proteins is hypothesized to result from postmortem proteolysis that generates soluble peptides. Desmin intermediate filaments play a well-characterized role in linking the myofibril to the costamere through associations with synemin and syncoilin (Capetanaki et al., 2007). Desmin filaments provide structural support and ensure appropriate alignment of adjacent myofibrils at the level of the Z-disk (Agnetti et al., 2022) and secure connection to the sarcolemma. Therefore, disruption of and degradation of desmin are expected to result in decreased muscle tissue integrity in postmortem meat.

Using a 2D DIGE approach, Carlson et al. (2017b) showed that a desmin degradation product associated with the desmin rod portion is soluble in a low-ionic-strength buffer. Desmin degradation products were more abundant in extracts from the more tender pork chops. In the current experiment, desmin was more abundant (adjusted *P* < 0.05; Tables 3 and 4) in Category A chops compared with the less tender chops in Categories C and D. Similarly, using immunoblotting (Figure 2), more desmin degradation product was identified in the low-ionic-strength fraction of chops in Category A than Category D. Johnson et al. (2023b) used this same library of samples to show less intact desmin in the myofibrillar fraction of tender chops and confirmed that observation with immunoblots. It is known that desmin is degraded during postmortem storage, that calpain-1 can catalyze that degradation (Huff-Lonergan et al., 1996a; Carlin et al., 2006), and that desmin degradation is associated with improved tenderization and water-holding capacity in fresh pork (Melody et al., 2004; Huff-Lonergan and Lonergan, 2005; Schulte et al., 2019, 2020). Qian et al. (2020) used calpain-specific and general proteinase inhibitors to show that inhibition of desmin degradation decreased drip channel formation and improved water-holding capacity in pork.

Degradation of desmin in postmortem muscle could increase the potential for extended proteolysis activated by calpain. Desmin filaments can anchor mitochondria near the myofibrils in living muscle (Capetanaki et al., 2007). Disruption of mitochondria structure and integrity likely begins with loosening the connection to the myofibril, perhaps resulting in loss of ability to sequester calcium. Decreased mitochondrial integrity has been linked to increased calcium in the sarcoplasm and activation of calpain-1 (Stafford et al., 2024).

Titin is the largest known polypeptide (~3–4 MDa) that spans half of the sarcomere with the amino terminus binding at the Z-disk and carboxy terminus at the M-Line (Meyer and Wright, 2013; Murphy et al., 2019). Titin is proposed to provide some architectural support to forming and established sarcomeres. Protein binding partners for titin include filamin, nebulin, obscurin, ankyrin, α-actinin, myomesin, calpain-1, and calpain-3 (reviewed by Kontrogioanni-Konstantopoulo et al., 2009). The size and diversity of binding partners make titin a critical player in muscle cell integrity. Titin (accession A0A480SN35) was more abundant (adjusted *P* < 0.05) in extracts from Category A chops than in all other categories. Titin (accession A0A480J3J1) was more abundant (adjusted *P* < 0.05) in Category A chops than chops in Categories C and D. Intact titin is insoluble, so the presence of these molecules in the low-ionic-strength buffer is proposed to be a result of proteolysis. Johnson et al. (2023b) showed that less titin was present in the myofibrillar fraction of Category A than in Category C or D chops, again suggesting the proteolytic release of titin fragments from the myofibrillar fraction. Degradation of titin is a common theme in the improvement of meat tenderness during the aging of beef (Rowe et al., 2004) and pork (Melody et al., 2004; Carlson et al., 2017a), and there is good evidence that calpain-1 can catalyze the reaction (Huff-Lonergan et al., 1996a, 1996b). The current results, taken with earlier observations, provide evidence that titin degradation products in the low-ionic-strength buffer could be reliable predictors of meat tenderness.

Filamin is a large (~290 kDa) actin-binding protein. In skeletal muscle, it is located at the periphery of the myofibril adjacent to the Z-disk, and it is bound to the intermediate filaments and the costamere in these regions (van der Flier and Sonnenberg., 2001), providing evidence that filamin provides architectural integrity within the muscle fiber. Chops in Category A had a greater abundance (adjusted *P* < 0.05) of filamin C in the low-ionic-strength extract than chops in every other category, thus making filamin C proteins in this soluble fraction an excellent candidate to predict tender pork. There is good evidence that filamin is degraded by calpain-1 (Huff-Lonergan et al., 1996; Du et al., 2023) and caspase-3 (Du et al., 2023). Filamin degradation is linked to improved tenderness in steaks from beef longissimus thoracis (Huff-Lonergan et al., 1996a; Wu et al., 2014) and pork loin chops (Carlson et al., 2017a). As postmortem aging progresses, a filamin degradation product (~278 kDa) is observed, generating a doublet with the intact product on a western blot. Carlson et al. (2017a) demonstrated that the degradation product included peptides from the entire span of the filamin protein except for the 109 amino acids at the carboxy-terminal end. This carboxy-terminal end is responsible for the dimerization of filamin and connection with other structural components, including integrin, portions of the potassium channels, and actin (Stossel et al., 2001; Feng and Walsh, 2004; Pudas et al., 2005; Nakamura et al., 2011). It is proposed that this degradation product would easily be released into the low-ionic-strength extraction buffer as it would no longer be tethered as a dimer associated with less soluble components with the cell and cellular components. Similarly, intact filamin should remain with the myofibrillar fraction. Therefore, proteolysis and tenderness should be linked to more filamin proteins in the low-ionic-strength extract. Johnson et al. (2023a) used this same library of samples to demonstrate a greater abundance of filamin C in the low-ionic-strength extract of chops with less purge loss, further linking postmortem changes of filamin with fresh pork quality traits.

Obscurin is a large (~720–900 kDa) muscle-specific protein that is part of the sarcomeric cytoskeleton (Manring et al., 2017) linked to the M-line and Z-disk that provides peripheral interaction with extrasarcomeric entities (Murphy et al., 2019). It uniquely contributes to the integrity of the myofibril connection to the sarcolemma and the sarcoplasmic reticulum. Obscurin localizes at the M-line due to its function to bind to myomesin. Notably, obscurin is not integrated with the myofibril but is closely connected (Kontrogianni-Konstantopoulos et al., 2009). It interacts directly with the ankyrin (Meyer and Wright, 2013; Manring et al., 2017) and is the only confirmed connection that aligns the sarcoplasmic reticulum with the myofibril. Specifically, obscurin organizes the longitudinal alignment of the sarcoplasmic reticulum along the myofibril (Lange et al., 2009). Obscurin is a unique gene product but has homology with titin. It binds to titin near the Z-disc and contributes to the connection of the myofibril to the sarcolemma through interaction with ankyrin molecules. The presence of obscurin molecules in the low-ionic-strength fraction implies disruption of these connections, perhaps through proteolysis. Johnson et al. (2023b) showed that the myofibrillar extract from Category A chops had less obscurin, suggesting migration of obscurin molecules to the low-ionic-strength soluble fraction, perhaps catalyzed by proteolysis. Very little is known regarding proteases that can degrade obscurin. A decrease in obscurin connections, by themselves, would not result in myofibrillar fragmentation but would result in decreased stability of the connection of myofibrils to the sarcolemma and a disruption of the connection to the sarcoplasmic reticulum that could leak sarcoplasmic reticulum contents, including calcium and proteins. Calsequestrin and sarcalumenin—key proteins within the sarcoplasmic reticulum—were more abundant (adjusted *P* < 0.05) in the extracts from Category A chops than Category C and D chops (Table 3), providing evidence of more disruption of the sarcoplasmic reticulum membrane in the more tender aged product.

Two gene products aligning with α-actinin were more abundant (adjusted *P* < 0.05) in Category A chops’ low-ionic-strength extracts than Category C or D chops (Table 3). It has long been held that α-actinin facilitates structural integrity in the Z-disk by cross-linking actin filaments (Suzuki et al., 1976). α-Actinin is not degraded in postmortem muscle, but Goll et al. (1991) demonstrated that exposure of myofibrils to calpains catalyzed a release of α-actinin from the myofibrillar fraction to a lower-ionic-strength fraction. More α-actinin in the low-ionic-strength extract in Category A in this study suggests more active proteolysis in the Category A chops. In a parallel study with myofibrillar extracts of the same pork chops, Johnson et al. (2023b) showed less α-actinin in Category A chops, thereby explaining a change in localization due to aging and proteolysis.

Junctophilins are transmembrane proteins with a transmembrane domain in the carboxy-terminal end. Junctophilins provide structural integrity between the T-tubule and the sarcoplasmic reticulum (Lehnart and Wehrens, 2022). They are essential to establish and support the triad structure in skeletal muscle (Tammineni et al., 2023). Human junctophilin-2 has calpain cleavage sites at residues 156, 204, and 572 (Wang et al., 2021), and the release of products at these sites could generate polypeptides that are soluble in the low-ionic-strength buffer as the highly insoluble sarcoplasmic reticulum membrane would no longer anchor them. Less (adjusted *P* < 0.05) junctophilin-1 was found in the soluble extract from Category A vs. D. The close proximity to the sarcoplasmic reticulum and the known susceptibility to degradation by calpain may make this protein less likely to be detected in aged products.

### Metabolic proteins

Metabolic enzymes and metabolites themselves can also directly affect the conversion of muscle to meat. The rate and extent of pH decline significantly alter pork tenderness and water-holding capacity. Initial pH decline, glycogen content, and substrate utilization in the glycolytic pathway are all critical factors in early postmortem muscle (England et al., 2014). Glycogen plays a pivotal role as a substrate for ATP production through glycogenolysis and glycolysis, influencing glycolytic potential (Mookerjee et al., 2016). The early postmortem meat environment is exceptionally dynamic, and this complicates efforts designed to define specific metabolic events or conditions that govern pH decline. Some evidence links phosphofructokinase activity, AMP deaminase activity, and mitochondrial intactness to pH decline (England et al., 2015; Matarneh et al., 2017). While the current results are in the context of aged pork, there is a different phenotype in the chops from the less tender Categories (D and C) than in Category A chops. Most notably, the aged chops in Category D had more sugars (adjusted *P* < 0.05), including mannose-6-phosphate, glucose-6-phosphate, fructose, glucose, and galactose. These metabolites remained after completion of rigor and persisted during aging, suggesting a difference in metabolism in muscle, early postmortem, and throughout the aging period. The proteomic profile of the soluble protein fraction in Category D illustrates a greater abundance of glycolytic enzymes, including glycogen debranching enzyme, α-enolase, phosphofructokinase, fructose-bisphosphate aldolase, and l-lactate dehydrogenase. An intriguing observation within the phosphagen system is a lesser abundance of adenylate kinase and a greater abundance of AMP deaminase in the protein extracts from Category A chops. Similarly, Johnson et al. (2023a) linked a greater abundance of AMP deaminase in the soluble fraction of chops with superior water-holding capacity. This combination in the early postmortem environment would promote less accumulation of AMP and presumably a lesser capacity to produce lactate (England et al., 2014). The chops in Category D had a lower pH than Category A chops (Supplementary Table 2), and there was a tendency (*P *= 0.051) for a greater amount of lactic acid in Category D chops (Table 2). Taken together, the Category D chops have a metabolomic profile that is consistent with muscle that emphasizes glycolytic metabolism.

### Antioxidant proteins

Oxidation and other modifications like glycation of proteins in postmortem muscle can influence protein functionality, activity, and structure. In postmortem muscle, these oxidative modifications can involve intra- and inter-protein cross-linking, oxidation of functional side chains, and adduction with lipid oxidation products (Lund et al., 2011). Numerous enzymes and structural proteins can be modified in response to oxidative stress and the environment in postmortem muscle. Lipid oxidation products modify muscle proteins like myoglobin (Suman et al., 2007; Nair et al., 2014), calpains (Zhai et al., 2023; Johnson et al., 2024), and lactate dehydrogenase (Ramanathan et al., 2014). In these cases, the functionality and/or solubility of the proteins was altered. Oxidation of the active site cysteine in calpain-1 promotes the formation of an intramolecular disulfide bond that inhibits calpain autolysis and activity (Lametsch et al., 2008; Maddock Carlin et al., 2024). Antioxidant proteins like peroxiredoxins have been shown to be more abundant in less tender meat (Carlson et al., 2017b; [Bibr CIT0050][Bibr CIT0079]), suggesting that antemortem oxidative stress may promote the production of these antioxidant proteins. In this study, peroxiredoxin-2 only tended (*P* = 0.09; Supplementary Table 4) to be more abundant in the Category D chops soluble fraction. Other antioxidant proteins—maillard deglycase (Tables 3 and 4) and hemopexin (Table 4)—were more abundant (adjusted *P* < 0.05) in the less tender chops. Maillard deglycase was also more abundant in the soluble protein fraction of Category C chops than the most tender Category A chops. Hemopexin has a role in binding to and clearing free heme iron in muscle, thereby limiting the possibility of oxidative damage (Karnaukhova et al., 2021). Patinho et al. (2024) reported a greater abundance of hemopexin at 9 h postmortem in beef demonstrating a high ultimate pH compared with normal pH beef. Maillard deglycase and peroxiredoxin-2 demonstrated more carbonylation modification in beef that was more tender (Malheiros et al., 2019). The roles of these proteins and the balance of oxidation and modification of other proteins in pork must be more precisely evaluated in the future.

### Chaperone proteins

Numerous heat shock proteins, including α-crystallin B chain, Galectin, and Heat Shock 27 kDa protein (Table 4), were more abundant (adjusted *P* < 0.05) in the low-ionic-strength extracts in Category A. Proteins in this class have diverse functions and causal relationships between the abundance of these proteins and meat tenderness are not clear (Purslow et al., 2021). However, several studies have provided some insight as these proteins can have antiapoptotic functions and could affect protein degradation (Gagaoua et al., 2015; Ma and Kim, 2020). Keeping these observations in context is essential as there could be differing effects considering species and muscle type. In beef, αβ-crystallin was more abundant in tender steaks from longissimus thoracis but less abundant in the tender steaks from the semitendinosus (Picard et al., 2014). Dang et al. (2022) demonstrated a greater abundance of chaperone proteins in beef steaks with intermediate tenderness compared with very tender steaks. The consistent association of heat shock proteins with meat quality traits is intriguing and points to important peri and antemortem metabolic events that influence fresh meat quality. These results in pork after a standard aging period suggest that heat shock proteins may be robust indicators of pork tenderness. Among those detected, α-crystallin B chain and Heat shock protein 27 demonstrated the greatest magnitude of difference with greater abundance in Category A. More direct investigations of the mechanisms by which these proteins contribute to differences in tenderness and overall quality are merited.

### Contextualizing the results

Bottom-up proteomic approaches have generated a great deal of data that can link proteomic profiles with specific phenotypes with a rapidity and efficiency that had never been achieved with other approaches. However, there is a limitation and a context to consider when evaluating results. Specifically, the nature of the peptide parent protein cannot be known, especially in postrigor meat, where the conditions are right for denaturation, modification, and degradation of proteins. Detecting unique peptides consistent with structural proteins not soluble in low-ionic-strength buffer illustrates the point. By fractionating the protein from aged pork chops, our approach can begin to contextualize the state of the parent protein. This is evident in the representative western blot (Figure 2A), where the nature of the parent protein of desmin identified with proteomics in the current study is markedly distinct from the parent protein of desmin from the myofibrillar fraction in our previous study (Johnson et al., 2023b). Identifying only desmin degradation products, which have routinely been linked to meat quality and tenderness, highlights the benefit of protein fractionation and is poised to connect the molecular state of a sample to its phenotypic quality traits.

The proteomic and metabolomic phenotypes defined were divergent across the tenderness (star probe) categories. The greatest number of differences in proteins and metabolites was noted in comparing the categories demonstrating the greatest differences in star probe. Category A chops were of high-quality, with slightly higher pH and lipid content and superior sensory tenderness scores. The molecular phenotype of the higher-quality aged chops illustrates a less glycolytic metabolism based on enzyme and substrate abundance. The abundance of glycolytic enzymes, the profile of the phosphagen system, and the persistent abundance of carbohydrates are consistent with the observed difference in 24 h and aged pH across Categories A and D. Although the rate of pH decline was not measured, the phenotype does suggest the rate of pH decline may have been slower in the Category A chops.

The observation of structural proteins in the low-ionic-strength protein extract is in agreement with previous observations that degradation products of desmin are soluble in these buffers that might be expected to contain sarcoplasmic proteins exclusively. Large and typically insoluble proteins known to be degraded in postmortem muscle, including titin, desmin, filamin, obscurin, and nebulin, were detected in these extracts with a greater abundance in the higher-quality Category A chops. Accumulation of degradation products is expected with these observations and a greater abundance of these products predicts more tender pork. Furthermore, a greater abundance of calsequestrin, calcium-transporting ATPases, and sarcalumenin implies a degree of disruption of membranes in the Category A chops. Therefore, the molecular phenotype of the higher-quality (Category A) aged chops is consistent with a slower and less extended pH decline, more disruption of membranes and membrane proteins, and degradation or release of structural proteins from the continuous connections of mitochondria, sarcoplasmic reticulum, myofibrils, and sarcolemma. This comprehensive study shows that the soluble protein fraction of aged pork is unique in that it contains numerous proteins that would not be expected to be in the soluble protein fraction in living muscle. The current interpretation is that the presence of these proteins is due to disruption of membranes, breakage of protein connections at the Z-disk, or release of soluble degradation products. These soluble fragments, derived from typically insoluble proteins, present valuable targets for molecular markers that could be harnessed to develop assays for predicting tenderness and other quality traits. The extent and rate at which these proteins or degradation products migrate to the soluble fraction postmortem remain unknown and require further definition. However, once characterized, these protein products could serve as valuable targets for assessing and predicting pork quality, ultimately enhancing its value.

## Supplementary Data

Supplementary data are available at *Journal of Animal Science* online.

skae355_suppl_Supplementary_Data

## Data Availability

The mass spectrometry proteomic and metabolomics data have been deposited in the MassIVE data repository (http://massive.ucsd.edu) with the dataset identifier MSV000095942.

## Abbreviations:

ACN: acetonitrile
AGC: automatic gain control
EDTA: ethylenediaminetetraacetic acid
FDR: false discovery rate
HCD: higher energy collisional dissociation
LC-MS/MS: liquid chromatography-tandem mass spectrometry
MeOH: methanol
MSTFA: *N*-methyl-*N*-(trimethylsilyl)trifluoroacetamide
SDS: sodium dodecyl sulfate
SDS-PAGE: sodium dodecyl sulfate-polyacrylamide gel electrophoresis
TEAB: triethyl ammonium bicarbonate
TMCS: 2,2,2-trifluoro-*N*-methyl-*N*-(trimethylsilyl)-acetamide, chlorotrimethylsilane
TMT: tandem mass tag
