# Supplementary material for: Proteomic and metabolomic profiling of aged pork loin chops reveals molecular phenotypes linked to pork tenderness
Source: J Anim Sci. 2024 Nov 20;102:skae355. doi: 10.1093/jas/skae355 (PMC11630860; doi:10.1093/jas/skae355)
Supplement: skae355_suppl_Supplementary_Data [file skae355_suppl_supplementary_data.zip › Supplemental_Information.docx]

Supplemental Information

**Proteomic and metabolomic features of aged fresh pork loin chops classified by instrumental tenderness**

Logan G. Johnson^*1^, Chaoyu Zhai^‡^, Kenneth J. Prusa^§^, Mahesh N. Nair^#^, Jessica E. Prenni^¶^ , Jacqueline M. Chaparro^¶^ Elisabeth Huff-Lonergan^*^, and Steven M. Lonergan^*1^

^*^Department of Animal Science, Iowa State University, Ames, Iowa 50011, USA

^§^Department of Food Science and Human Nutrition, Iowa State University, Ames, Iowa 50011, USA

^#^Department of Animal Sciences, Colorado State University, Fort Collins, Colorado 80523, USA

^‡^Department of Animal Science, University of Connecticut, Storrs, Connecticut 06269-4040, USA

^¶^Department of Horticulture and Landscape Architecture, Colorado State University, Fort Collins, CO 80523, USA

^1^Corresponding author: slonerga@iastate.edu

Author Lonergan is a Section Editor for the Journal of Animal Science. Author Huff-Lonergan is the Editor in Chief for the Journal of Animal Science. Both authors have recused themselves from the review process.

**Supplementary Table 1.** Summary of pork quality traits from the initial population of 120 (*N* = 120) commercial pork loins. (from Johnson et al. 2023b)

| **Attribute** | **Range** | **Mean** | **Median** | **Standard Deviation** |
| --- | --- | --- | --- | --- |
| Star Probe, kg^1^ | 3.43 – 7.41 | 5.16 | 5.16 | 0.77 |
| 24 h pH | 5.51 – 6.01 | 5.68 | 5.68 | 0.10 |
| Aged pH | 5.62 – 6.07 | 5.78 | 5.77 | 0.09 |
| Loin Purge, % | 0.00 – 2.73 | 0.61 | 0.44 | 0.57 |
| Chop Purge, % | 0.06 – 1.86 | 0.74 | 0.70 | 0.34 |
| Moisture Content, % | 72.97 – 78.15 | 74.83 | 74.66 | 0.93 |
| Lipid Content, % | 0.83 – 4.86 | 2.03 | 1.88 | 0.76 |
| Marbling Score^2^ | 1.0 – 4.0 | 2.0 | 2.0 | 0.6 |
| Color Score^3^ | 2.0 – 4.5 | 3.2 | 3.0 | 0.6 |
| Aged *L**^4^ | 42.24 – 54.07 | 48.26 | 48.24 | 2.26 |
| Cook Loss, % | 15.57 – 27.74 | 22.49 | 22.71 | 2.45 |
| Tenderness^5^ | 4.0 – 10.0 | 6.8 | 7.0 | 1.1 |
| Chewiness^5^ | 1.0 – 6.0 | 3.2 | 3.0 | 0.9 |
| Juiciness^5^ | 5.0 – 9.0 | 6.6 | 7.0 | 0.9 |
| Flavor^5^ | 2.0 – 8.0 | 4.4 | 4.0 | 1.1 |
| Off Flavor^5^ | 1.0 – 5.0 | 1.7 | 2.0 | 0.8 |

^1^A 5-point star probe attachment fitted with an Instron was used to assess the force needed to compress a chop to 20% of its original height (Carlson et al., 2017b).

^2^National Pork Board standards, 10-point scale (1 = 1% intramuscular fat; 10 = 10% intramuscular fat).

^3^National Pork Board standards, 6-point scale (1 = pale pinkish gray/white; 6 = dark purplish red). ^4^Hunter L* determined with Minolta Chroma ^4^Meter with D65 light source, 50 mm aperture, and 2° observer.

^5^As determined by a trained panel (*N* = 4) using a 10-point category scale.

**Supplementary Table 2.** Summary of pork quality and sensory attributes from aged pork loins classified by instrumental star probe group. (Table 1 in Johnson et al. 2023b)

| **Attribute** | **Category A^1^**  **(*n* = 25)** | **Category B^1^**  **(*n* = 25)** | **Category C^1^**  **(*n* = 25)** | **Category D^*^**  **(*n* = 25)** | **SEM** | **Star Probe Category *P*-value** | **Sex  *P-*value** | **Sire line *P*-value** | **Slaughter Date  *P*-value** |
| --- | --- | --- | --- | --- | --- | --- | --- | --- | --- |
| Star Probe, kg^1^ | 4.23^d^ | 4.79^c^ | 5.43^b^ | 6.21^a^ | 0.06 | < 0.01 | < 0.01 | 0.25 | 0.96 |
| 24 h pH | 5.75^a^ | 5.70^ab^ | 5.65^bc^ | 5.63^c^ | 0.02 | < 0.01 | 0.36 | 0.07 | 0.05 |
| Aged pH | 5.84^a^ | 5.79^b^ | 5.75^bc^ | 5.73^c^ | 0.02 | < 0.01 | 0.32 | < 0.01 | 0.90 |
| Loin Purge, % | 0.32^c^ | 0.50^bc^ | 0.90^ab^ | 0.75^a^ | 0.12 | < 0.01 | 0.51 | 0.06 | 0.13 |
| Chop Purge, % | 0.58 | 0.73 | 0.83 | 0.83 | 0.07 | 0.06 | 0.81 | 0.02 | 0.37 |
| Moisture Content, % | 74.71 | 74.68 | 74.81 | 74.81 | 0.18 | 0.92 | 0.12 | 0.03 | 0.44 |
| Lipid Content, % | 2.39^a^ | 1.96^b^ | 1.96^b^ | 1.86^b^ | 0.14 | 0.07 | 0.02 | < 0.01 | 0.63 |
| Marbling Score^2^ | 2.4^a^ | 2.0^b^ | 2.1^b^ | 1.8^b^ | 0.1 | 0.01 | < 0.01 | < 0.01 | 0.10 |
| Color Score^3^ | 3.5^a^ | 3.2^ab^ | 3.1^bc^ | 2.9^c^ | 0.1 | < 0.01 | 0.03 | 0.01 | 0.07 |
| Aged *L**^4^ | 47.74^b^ | 47.66^ab^ | 48.20^a^ | 49.32^a^ | 0.43 | < 0.05 | 0.97 | 0.02 | 0.03 |
| Cook Loss, % | 21.26^b^ | 22.34^b^ | 22.87^ab^ | 23.54^a^ | 0.53 | < 0.05 | 0.76 | 0.84 | 0.07 |
| Tenderness^5^ | 7.6^a^ | 7.1^a^ | 6.4^b^ | 6.3^b^ | 0.2 | < 0.01 | 0.12 | 0.92 | < 0.01 |
| Chewiness^5^ | 2.5^c^ | 3.1^b^ | 3.5^ab^ | 3.9^a^ | 0.2 | < 0.01 | 0.62 | 0.10 | 0.01 |
| Juiciness^5^ | 6.8^a^ | 6.7^ab^ | 6.4^ab^ | 6.2^b^ | 0.2 | 0.13 | 0.36 | 0.55 | 0.01 |
| Flavor^5^ | 5.5^a^ | 4.5^b^ | 4.2^b^ | 3.6^c^ | 0.2 | < 0.01 | 0.78 | 0.06 | 0.10 |
| Off Flavor^5^ | 1.3^b^ | 1.5^b^ | 1.7^b^ | 2.3^a^ | 0.1 | < 0.01 | 0.59 | < 0.05 | 0.10 |
| 55-kDa Intact Desmin in the myofibrillar fraction^6^ | 0.47^b^ | – | – | 0.94^a^ | 0.05 | < 0.01 | – | – | – |

^1^A 5-point star probe attachment fitted with an Instron was used to assess the force needed to compress a chop to 20% of its original height (Carlson et al., 2017b).

^2^National Pork Board standards, 10-point scale (1 = 1% intramuscular fat; 10 = 10% intramuscular fat).

^3^National Pork Board standards, 6-point scale (1 = pale pinkish gray/white; 6 = dark purplish red).

^4^Hunter L* determined with Minolta Chroma Meter with D65 light source, 50 mm aperture, and 2° observer.

^5^As determined by a trained panel (*N* = 4) using a 10-point category scale.

^6^Ratio of the densitometry units of the intact 55-kDa desmin band in the myofibrillar fraction of the sample over the intact 55-kDa desmin band of the reference sample.

^a,b,c,d^Means within rows with different superscripts are significantly different (*P* < 0.05).

^*^From the distribution of star probe values of the initial population of pork loins (*N* = 120), chops in Group A represent the lowest 0–20% in value, Group B are the next highest 25–45%, Group C are the next 55–75%, and Group D are the highest 80–100%.

**Supplemental Table 3.** Molecules detected but not different between Categories A vs. D^1,2^

|  | **Log_2_ Fold Difference** | | **Adjusted P values** | |
| --- | --- | --- | --- | --- |
|  | **A vs. C** | **A vs. D** | **A vs. C** | **A vs. D** |
|  |  |  |  |  |
| Glycine | 0.17 | 0.10 | 0.02 | 0.12 |
| Glyceric Acid | 0.59 | 0.25 | 0.02 | 0.29 |
| Hypotaurine | 0.24 | 0.04 | 0.03 | 0.70 |
| Octadecadienocic Acid | 1.12 | 0.61 | 0.06 | 0.27 |
| Hexadecanoic Acid | 0.67 | 0.21 | 0.06 | 0.50 |
| Urea | 0.16 | 0.03 | 0.11 | 0.73 |
| Glycerol-3-phosphate | 0.28 | 0.31 | 0.12 | 0.06 |
| Sarcosine | 0.11 | 0.11 | 0.22 | 0.15 |
| Pyroglutamic acid | 0.11 | 0.08 | 0.24 | 0.33 |
| Oxalic acid | 0.12 | -0.11 | 0.25 | 0.27 |
| Alanine | 0.16 | 0.11 | 0.26 | 0.38 |
| Arabitol (sugar alcohol) | 0.16 | 0.19 | 0.27 | 0.15 |
| Adenosine-5-monophosphate | 0.14 | -0.02 | 0.27 | 0.89 |
| Lumichrome | -2.00 | -0.20 | 0.28 | 0.08 |
| Myo-inositol | -0.13 | -0.06 | 0.33 | 0.67 |
| Asparagine | 0.09 | -0.07 | 0.37 | 0.46 |
| Ribose | -0.05 | -0.11 | 0.59 | 0.23 |
| Creatinine | -0.02 | -0.16 | 0.86 | 0.06 |
| Inosine | -0.02 | -0.29 | 0.86 | 0.07 |
| Nicotinamide | 0.02 | -0.13 | 0.86 | 0.10 |
| Phosphoric acid | 0.02 | -0.11 | 0.86 | 0.26 |

^1^Categories A, C, and D were classified by instrumental star probe tenderness. Category A (n = 25): $\bar{x}$ =4.23 kg*,* 3.43-4.55 kg; Category B (n = 25): $\bar{x}$= 4.79 kg, 4.66-5.00 kg; Category C (n = 23): $\bar{x}$ = 5.43 kg, 5.20-5.64 kg; Category D (n =24): $\bar{x}$ = 6.21 kg, 5.70-7.41.

^2^No differences in identified metabolites were detected between Categories A and B, Categories B and C, or Categories C and D.

**Supplemental Table 4.** Summary of proteins detected and included in the analysis (present in more than half of the samples, more than 1 unique peptide) that were not affected by category

| **Accession Number** | **Description** | **A vs. D**  **Log2 Fold Difference** | **A vs. D**  **Adjusted**  **P-value** |
| --- | --- | --- | --- |
| A0A481CLJ7 | 1,2-dihydroxy-3-keto-5-methylthiopentene dioxygenase (Fragment) | -0.232 | 0.236 |
| P59083 | 14 kDa phosphohistidine phosphatase | -0.080 | 0.334 |
| A0A480LG53 | 14-3-3 protein beta/alpha (Fragment) | 0.158 | 0.856 |
| A0A480PLY3 | 14-3-3 protein zeta/delta | 0.201 | 0.657 |
| A0A286ZSB5 | 15S Mg(2+)-ATPase p97 subunit | 0.012 | 0.918 |
| A0A4X1UZP1 | 2-phospho-D-glycerate hydro-lyase | -0.035 | 0.738 |
| A0A4X1TPH9 | 3-hydroxyisobutyrate dehydrogenase, mitochondrial | 0.125 | 0.811 |
| F1S232 | 4-trimethylaminobutyraldehyde dehydrogenase | -0.030 | 0.811 |
| K7GPB8 | 5'-nucleotidase domain containing 1 | 0.015 | 0.965 |
| A0A4X1SP24 | 5'-nucleotidase | -0.308 | 0.424 |
| F1SMZ7 | 60 kDa chaperonin | 0.065 | 0.441 |
| F1RGJ3 | 75 kDa glucose-regulated protein | 0.076 | 0.595 |
| A0A480UES1 | 78 kDa glucose-regulated protein | -0.026 | 0.857 |
| A0A4X1U5J5 | AA_TRNA_LIGASE_II domain-containing protein | -0.032 | 0.941 |
| A0A4X1UIN8 | Acid phosphatase | 0.127 | 0.690 |
| A0A0B8RTA5 | Aconitate hydratase, mitochondrial | -0.030 | 0.775 |
| A0A287A6P1 | Actin-depolymerizing factor | 0.123 | 0.858 |
| A0A4X1SLV1 | Acyl-CoA-binding protein | -0.085 | 0.370 |
| P00819 | Acylphosphatase-2 | -0.047 | 0.660 |
| A0A287B4J2 | Adenosylhomocysteinase | 0.067 | 0.606 |
| A0A5G2QNB5 | Adenylate kinase 2, mitochondrial | 0.062 | 0.871 |
| A0A4X1TTC3 | Adenylosuccinate synthetase isozyme 1 | 0.064 | 0.532 |
| R4H1Z8 | Adipocyte-type fatty acid-binding protein | 0.122 | 0.382 |
| Q6Q2K6 | Adiponectin (Fragment) | 0.013 | 0.937 |
| A0A286ZT13 | Albumin | -0.060 | 0.674 |
| A0A481ARR7 | Aldehyde dehydrogenase (NAD(+)) | -0.012 | 0.957 |
| A0A5G2RA17 | Aldehyde dehydrogenase 6 family member A1 | -0.393 | 0.415 |
| A0A4X1VTJ3 | Aldo-keto reductase family 1 member B1 | -0.090 | 0.308 |
| Q29014 | Alpha-1 acid glycoprotein (Fragment) | -0.158 | 0.349 |
| A0A4X1V5H0 | Alpha-1,4 glucan phosphorylase | -0.228 | 0.309 |
| A0A4X1SEW9 | Alpha-1,4 glucan phosphorylase | 0.083 | 0.888 |
| A0A480EDG3 | Alpha-protein kinase 3 | 0.255 | 0.352 |
| K7GQF6 | Aminopeptidase like 1 | -0.145 | 0.685 |
| A0A5G2R745 | Aminopeptidase | -0.081 | 0.258 |
| P19620 | Annexin A2 | -0.103 | 0.441 |
| **Accession Number** | **Description** | **A vs. D**  **Log2 Fold Difference** | **A vs. D**  **Adjusted**  **P-value** |
| F1SJB5 | Annexin | -0.094 | 0.757 |
| A0A4X1VBD0 | Annexin | -0.211 | 0.205 |
| A0A4X1TMS8 | Annexin | -0.144 | 0.078 |
| K7GM40 | Apolipoprotein A-I | -0.065 | 0.650 |
| P00503 | Aspartate aminotransferase, cytoplasmic | 0.096 | 0.676 |
| P00506 | Aspartate aminotransferase, mitochondrial | 0.037 | 0.746 |
| A0A4X1TR60 | ATP synthase F1 subunit epsilon | 0.034 | 0.939 |
| A0A287AGU2 | ATP synthase subunit alpha | 0.151 | 0.677 |
| A0A4X1U774 | ATP-dependent (S)-NAD(P)H-hydrate dehydratase | -0.086 | 0.835 |
| A0A4X1T6H5 | ATP-grasp domain-containing protein | -0.145 | 0.771 |
| A0A0B8RTW8 | BCL2-associated athanogene 3 | -0.078 | 0.234 |
| A0A4X1TVC9 | Bleomycin hydrolase | -0.114 | 0.408 |
| A0A4X1VMP3 | BTB domain-containing protein | 0.100 | 0.474 |
| A0A4X1TK38 | BTB domain-containing protein | 0.138 | 0.594 |
| F1SSR4 | Butyryl-CoA dehydrogenase | -0.141 | 0.833 |
| F1SRC8 | C-type lectin domain family 3 member B | -0.093 | 0.549 |
| A0A5G2QF74 | Cadherin-13 | -0.123 | 0.568 |
| A0A287AUZ3 | Calcium binding protein 39 | -0.038 | 0.957 |
| A0A480EV23 | Calcium-activated neutral proteinase 1 | -0.008 | 0.957 |
| I3LQD3 | Calcium-activated neutral proteinase 2 | 0.029 | 0.921 |
| B5L0Y6 | Calpain inhibitor | -0.262 | 0.302 |
| P04574 | Calpain small subunit 1 | 0.057 | 0.786 |
| A0A481CNC7 | Calpain-3 | 0.014 | 0.924 |
| P28491 | Calreticulin | -0.097 | 0.365 |
| F1SD45 | cAMP-dependent protein kinase | -0.139 | 0.160 |
| A0A287AKY1 | cAMP-dependent protein kinase type I-alpha regulatory subunit | -0.172 | 0.134 |
| A0A4X1T087 | cAMP-dependent protein kinase type II-alpha regulatory subunit | -0.089 | 0.771 |
| Q5S1S4 | Carbonic anhydrase 3 | -0.082 | 0.820 |
| A0A0K1TQQ7 | Carbonyl reductase 3 | 0.094 | 0.572 |
| B8LFE3 | Cardiomyopathy associated 1 | 0.024 | 0.896 |
| F1SGS9 | Catalase | -0.031 | 0.957 |
| A0A4X1URU2 | Catechol O-methyltransferase | -0.495 | 0.270 |
| A0A5G2R9Y3 | CBM20 domain-containing protein | 0.145 | 0.369 |
| A0A5G2QMG1 | CCT-beta | 0.249 | 0.171 |
| Q007T2 | Cell division control protein 42 homolog | 0.098 | 0.416 |
| I3VKE6 | Ceruloplasmin | 0.168 | 0.619 |
| B2CZR7 | CFL2b variant 1 | 0.070 | 0.677 |
| **Accession Number** | **Description** | **A vs. D**  **Log2 Fold Difference** | **A vs. D**  **Adjusted**  **P-value** |
| A0A0B8S0A2 | Chloride intracellular channel protein | 0.035 | 0.957 |
| P00889 | Citrate synthase, mitochondrial | -0.165 | 0.072 |
| A0A4X1SNG6 | CMP/dCMP-type deaminase domain-containing protein | -0.418 | 0.173 |
| A0A4X1UWI9 | CN hydrolase domain-containing protein | -0.097 | 0.893 |
| A0A0D5BWD2 | Complement component 1 Q subcomponent-binding protein, mitochondrial | -0.041 | 0.897 |
| A7TX82 | COP9 signalosome complex subunit 2 | 0.158 | 0.594 |
| A0A5G2QP70 | COP9 signalosome complex subunit 3 | 0.052 | 0.866 |
| A7Y521 | COP9 signalosome complex subunit 4 | -0.017 | 0.955 |
| A0A4X1T9H3 | COP9 signalosome complex subunit 8 | -0.102 | 0.674 |
| A0A4X1TZM5 | Coronin | 0.007 | 0.986 |
| Q5XLD3 | Creatine kinase M-type | -0.050 | 0.856 |
| A0A4X1TV43 | Creatine kinase | 0.040 | 0.894 |
| Q2HYU1 | Creatine kinase | -0.019 | 0.893 |
| A0A287ARP6 | CYFIP related Rac1 interactor B | -0.291 | 0.360 |
| F6Q6A7 | Cysteine and glycine rich protein 3 | 0.068 | 0.810 |
| P62895 | Cytochrome c | 0.268 | 0.072 |
| F1SGG3 | Cytokeratin-1 | -0.067 | 0.888 |
| P28839 | Cytosol aminopeptidase | -0.013 | 0.932 |
| A0A4X1T5Y1 | Diadenosine tetraphosphate synthetase | -0.163 | 0.638 |
| P09623 | Dihydrolipoyl dehydrogenase, mitochondrial | 0.089 | 0.324 |
| Q9N0F1 | Dihydrolipoyllysine-residue succinyltransferase component of 2-oxoglutarate dehydrogenase complex, mitochondrial | 0.209 | 0.261 |
| F1RU52 | Dipeptidyl peptidase 3 | 0.015 | 0.924 |
| A0A4X1VLC3 | DNA damage-binding protein 1 | 0.236 | 0.641 |
| A0A4X1TQI1 | Dual specificity protein phosphatase | -0.154 | 0.096 |
| A0A4X1SKB8 | Dynein light chain | -0.099 | 0.850 |
| Q5GN48 | Dystrophin | -0.028 | 0.850 |
| A0A4X1UY91 | eIF-5a domain-containing protein | 0.034 | 0.905 |
| Q6UAQ9 | Electron transfer flavoprotein subunit alpha, mitochondrial (Fragment) | -0.059 | 0.606 |
| A0A5G2R0H0 | Electron transfer flavoprotein subunit beta | -0.103 | 0.369 |
| A0A4X1SRH0 | Elongation factor 1-alpha | 0.126 | 0.172 |
| F1SHD6 | Elongation factor 1-beta | -0.109 | 0.568 |
| Q29387 | Elongation factor 1-gamma (Fragment) | -0.012 | 0.913 |
| A0A287AWI9 | Elongation factor 2 | 0.079 | 0.190 |
| A0A480JRE1 | Enoyl-CoA hydratase, mitochondrial | 0.100 | 0.365 |
| A0A4X1VD81 | Eukaryotic translation initiation factor 3 30 kDa subunit | 0.422 | 0.474 |
| A0A5G2RC36 | Eukaryotic translation initiation factor 4E | 0.098 | 0.833 |
| **Accession Number** | **Description** | **A vs. D**  **Log2 Fold Difference** | **A vs. D**  **Adjusted**  **P-value** |
| C0LZL1 | Fascin | 0.017 | 0.956 |
| Q68S99 | Fatty acid binding protein 3 (Fragment) | 0.002 | 0.986 |
| F6PVY0 | Fatty acid binding protein 5 | -0.092 | 0.327 |
| F1RX35 | Fibrinogen gamma chain | 0.384 | 0.354 |
| A0A4X1T9I2 | Gc-globulin | 0.150 | 0.442 |
| A0A287BG92 | Geranylgeranyl transferase type-2 subunit beta | 0.085 | 0.814 |
| A0A480S836 | Glucose-6-phosphate isomerase | 0.059 | 0.871 |
| A0A4X1VHE8 | Glutamate dehydrogenase (NAD(P)(+)) | 0.212 | 0.345 |
| A0A5G2QQ71 | Glutaredoxin 3 | -0.173 | 0.365 |
| P12309 | Glutaredoxin-1 | -0.092 | 0.857 |
| A0A4X1TEC7 | Glutathione S-transferase | 0.155 | 0.441 |
| A0A480HNH3 | Glutathione-dependent dehydroascorbate reductase | -0.106 | 0.455 |
| G3CKJ2 | Glyceraldehyde-3-phosphate dehydrogenase (Fragment) | 0.254 | 0.163 |
| A0SNU7 | Glyceraldehyde-3-phosphate dehydrogenase (Fragment) | 0.022 | 0.957 |
| A0A4X1UVA2 | Glyceraldehyde-3-phosphate dehydrogenase | 0.096 | 0.675 |
| I3LLU0 | Glycerol-3-phosphate dehydrogenase [NAD(+)] | -0.437 | 0.490 |
| A0A480PWJ1 | Glycogenin-1 isoform 1 | -0.108 | 0.213 |
| A0A4X1U3R7 | Glyoxalase domain-containing protein 4 | -0.116 | 0.345 |
| A0A480PNH9 | GMP reductase | -0.244 | 0.143 |
| B6E241 | Growth factor receptor bound protein 2 | -0.042 | 0.866 |
| A0A5G2QZY6 | GTP-binding nuclear protein Ran | 0.264 | 0.207 |
| Q5PYH3 | GTP-binding protein SAR1b | -0.318 | 0.354 |
| A0A4X1VP82 | Guanidinoacetate N-methyltransferase | -0.005 | 0.979 |
| A0A287B6J6 | Heat shock 70 kDa protein 4 | 0.015 | 0.932 |
| A5A8V6 | Heat shock 70kDa protein 1A | 0.079 | 0.457 |
| A0A4X1TS37 | Heat shock protein HSP 90-alpha | 0.017 | 0.895 |
| P01965 | Hemoglobin subunit alpha | -0.121 | 0.349 |
| A0A4X1SGD5 | Hemopexin | -0.148 | 0.356 |
| A0A287ATP0 | Heparan sulfate proteoglycan 2 | 0.287 | 0.382 |
| F1RKI3 | Histidine triad nucleotide binding protein 1 | -0.009 | 0.957 |
| A0A4X1TUA6 | HMA domain-containing protein | -0.235 | 0.408 |
| Q684M6 | Hsp90 chaperone protein kinase-targeting subunit | 0.030 | 0.957 |
| A0A4X1UT70 | Hydroxyacyl-coenzyme A dehydrogenase, mitochondrial | -0.062 | 0.638 |
| F1SGI7 | IF rod domain-containing protein | -0.467 | 0.354 |
| A0A4X1UKQ2 | IF rod domain-containing protein | 0.154 | 0.136 |
| A0A4X1WBK5 | IF rod domain-containing protein | -0.205 | 0.685 |
| A0A4X1W641 | IF rod domain-containing protein | -0.391 | 0.480 |
| **Accession Number** | **Description** | **A vs. D**  **Log2 Fold Difference** | **A vs. D**  **Adjusted**  **P-value** |
| A0A075B7H9 | Ig-like domain-containing protein | 0.143 | 0.328 |
| L8B0W0 | IgG heavy chain | -0.199 | 0.536 |
| I3LUI1 | Immunoglobulin like and fibronectin type III domain containing 1 | -0.123 | 0.550 |
| A0A5G2R9L2 | Importin subunit alpha | -0.097 | 0.835 |
| F1RWJ5 | Importin subunit beta-1 | 0.215 | 0.328 |
| A0A480TTD3 | Importin-5 isoform X1 | 0.469 | 0.159 |
| F1SUE3 | Inorganic diphosphatase | -0.001 | 0.995 |
| A0A4X1SXS6 | Inosine-5'-monophosphate dehydrogenase | 0.342 | 0.568 |
| A0A5G2Q9Z0 | Inositol-1-monophosphatase | -0.005 | 0.967 |
| A0A287A8M1 | Isocitrate dehydrogenase [NADP] | -0.337 | 0.267 |
| A0A4X1UPE2 | J domain-containing protein | 0.020 | 0.957 |
| A0A4X1TUL5 | Junctophilin | 0.192 | 0.126 |
| I3LDS3 | Keratin 10 | -0.441 | 0.245 |
| P00336 | L-lactate dehydrogenase B chain | -0.151 | 0.070 |
| A0A4X1SRH7 | Lactoylglutathione lyase | -0.137 | 0.159 |
| A0A5G2QKZ6 | Laminin subunit beta 1 | -0.092 | 0.709 |
| F1S663 | Laminin subunit gamma 1 | -0.016 | 0.957 |
| A0A287B283 | LanC like 1 | -0.048 | 0.765 |
| A0A480TXA1 | Leucine-rich repeat-containing protein 20 isoform 1 (Fragment) | 0.122 | 0.857 |
| A0A5G2Q8A7 | LIM and cysteine-rich domains protein 1 | 0.086 | 0.503 |
| A0A480K7Y0 | LIM domain-binding protein 3 | -0.664 | 0.108 |
| A0A480PCQ6 | Long-chain specific acyl-CoA dehydrogenase, mitochondrial | 0.061 | 0.833 |
| A0A4X1SG78 | Lumican | -0.051 | 0.672 |
| A0A5G2QFC2 | Malate dehydrogenase | -0.137 | 0.366 |
| A0A5G2RGL7 | Malate dehydrogenase | -0.137 | 0.060 |
| F1RQM4 | Malic enzyme | -0.094 | 0.550 |
| A0A4X1TRT9 | Mannose-6-phosphate isomerase | -0.161 | 0.361 |
| A0A480MV49 | Metavinculin | -0.037 | 0.715 |
| A0A4X1SQ67 | Methylthioribulose-1-phosphate dehydratase | 0.131 | 0.674 |
| A0A287AY86 | Microtubule-associated protein | -0.040 | 0.846 |
| A0A287AKE9 | Microtubule-associated protein RP/EB family member 2 | 0.200 | 0.196 |
| A0A4X1TYW8 | Mimecan | -0.103 | 0.893 |
| M9TGS8 | Mitochondrial delta3,delta2-dienoyl-CoA isomerase | 0.008 | 0.957 |
| A0A1W6R2B4 | Mitsugumin-53 | 0.051 | 0.800 |
| A0A4X1W5I7 | Multifunctional fusion protein | 0.306 | 0.267 |
| A0A4X1VEW6 | Muscle-restricted coiled-coil protein | 0.003 | 0.990 |
| K4EJ64 | MYL3 | -0.205 | 0.145 |
| **Accession Number** | **Description** | **A vs. D**  **Log2 Fold Difference** | **A vs. D**  **Adjusted**  **P-value** |
| P02189 | Myoglobin | -0.152 | 0.660 |
| A0A5G2RCH7 | Myomesin 1 | -0.049 | 0.606 |
| A0A480EA26 | Myopalladin isoform a | 0.241 | 0.354 |
| A0A5G2QM90 | Myosin binding protein C1 | 0.380 | 0.521 |
| Q9TV62 | Myosin-4 | 0.144 | 0.207 |
| A0A480TFS2 | Myosin-binding protein H | -0.056 | 0.811 |
| K9IVW4 | Myotrophin | -0.067 | 0.527 |
| A0A287BPK4 | Myozenin 3 | -0.102 | 0.765 |
| Q4PS85 | Myozenin-1 | 0.174 | 0.207 |
| A0A480XYA6 | N-acyl-L-amino-acid amidohydrolase | -0.005 | 0.967 |
| A0A4X1SPS1 | NAD(P)-bd_dom domain-containing protein | -0.108 | 0.704 |
| A0A5G2QPA8 | NAD(P)H-hydrate epimerase | -0.108 | 0.685 |
| A0A480EUT8 | Nascent polypeptide-associated complex subunit alpha isoform X2 | -0.050 | 0.572 |
| A0A480Z467 | Neuroblast differentiation-associated protein AHNAK isoform X1 | 0.313 | 0.213 |
| A0A480NKX9 | NHL repeat-containing protein 2 | -0.229 | 0.328 |
| F1RGY5 | Nidogen 1 | 0.031 | 0.926 |
| A0A5G2QWH5 | NIPSNAP domain-containing protein | 0.109 | 0.765 |
| A0A481D0P1 | Nucleoside diphosphate kinase (Fragment) | -0.158 | 0.334 |
| A0A480NF45 | Obscurin isoform b | 0.153 | 0.297 |
| K9IVI1 | Oxoglutarate dehydrogenase (succinyl-transferring) | 0.303 | 0.595 |
| C1L369 | Parvalbumin | -0.394 | 0.136 |
| Q6QGC0 | PDZ and LIM domain protein 3 | -0.087 | 0.375 |
| A0A4X1V2S2 | Peptidase S1 domain-containing protein | -0.050 | 0.677 |
| P62936 | Peptidyl-prolyl cis-trans isomerase A | -0.141 | 0.120 |
| I3LR51 | Peptidylprolyl isomerase | 0.083 | 0.533 |
| F1S418 | Peroxiredoxin 3 | -0.135 | 0.709 |
| A0A287AJ76 | Peroxiredoxin-2 | -0.152 | 0.094 |
| Q9TSX9 | Peroxiredoxin-6 | -0.016 | 0.878 |
| A0A481DN55 | Phosphoacetylglucosamine mutase | 0.128 | 0.354 |
| G0Z3A1 | Phosphoglucomutase 1 | -0.192 | 0.650 |
| A0A4X1VTD9 | Phosphoglycerate mutase | -0.049 | 0.666 |
| A0A287AJQ2 | Phosphoglycerate mutase | 0.111 | 0.213 |
| A0A287BDT8 | Phosphoglycolate phosphatase | 0.053 | 0.808 |
| A0A4X1UIS9 | Phosphoinositide phospholipase C | 0.228 | 0.213 |
| A0A4X1SS40 | Phosphorylase b kinase regulatory subunit | 0.710 | 0.172 |
| A0A5G2QDJ2 | Polyubiquitin-C | -0.051 | 0.674 |
| A0A5G2R9A9 | Profilin | -0.111 | 0.322 |
| **Accession Number** | **Description** | **A vs. D**  **Log2 Fold Difference** | **A vs. D**  **Adjusted**  **P-value** |
| A0A480KNJ7 | Programmed cell death 6-interacting protein isoform 1 | -0.036 | 0.835 |
| A0A287AZX9 | Prolyl endopeptidase | -0.180 | 0.550 |
| A0A5G2QTM2 | Prostaglandin E synthase 3 | 0.131 | 0.733 |
| A0A5G2QTK7 | Proteasome subunit alpha type | 0.027 | 0.867 |
| F2Z5L7 | Proteasome subunit alpha type | 0.118 | 0.334 |
| I3LAB6 | Proteasome subunit alpha type | -0.136 | 0.474 |
| A0A5G2R2Y1 | Proteasome subunit alpha type | -0.041 | 0.808 |
| A0A5G2QL81 | Proteasome subunit alpha type | -0.069 | 0.375 |
| A0A480SCD0 | Proteasome subunit alpha type | -0.231 | 0.141 |
| A0A4X1UXP5 | Proteasome subunit alpha type | -0.037 | 0.902 |
| A0A4X1V3T5 | Proteasome subunit beta | 0.029 | 0.895 |
| A0A4X1UYL6 | Proteasome subunit beta | 0.022 | 0.918 |
| A0A480LMY7 | Proteasome subunit beta | -0.047 | 0.850 |
| A0A481A772 | Proteasome subunit beta | -0.015 | 0.940 |
| A1XQU1 | Proteasome subunit beta type-7 | -0.180 | 0.144 |
| A0A5G2QA32 | Protein arginine methyltransferase 1 | -0.064 | 0.811 |
| E1CAJ5 | Protein disulfide-isomerase | 0.010 | 0.957 |
| A0A4X1UBN3 | Protein disulfide-isomerase | -0.053 | 0.808 |
| F1SAD9 | Protein disulfide-isomerase | 0.100 | 0.856 |
| A0A287AR93 | Protein kinase C and casein kinase substrate in neurons 3 | 0.107 | 0.870 |
| A0A4X1UXH8 | Protein NDRG2 | -0.213 | 0.067 |
| A0A480Q2M0 | Protein phosphatase 1 regulatory subunit 1A (Fragment) | -0.102 | 0.559 |
| F1SJC2 | Protein phosphatase 1 regulatory subunit 3A | -0.228 | 0.108 |
| P80895 | Protein-L-isoaspartate(D-aspartate) O-methyltransferase | -0.128 | 0.302 |
| A0A5S8KL32 | Protein-serine/threonine phosphatase | -0.004 | 0.985 |
| A0A4X1VZD7 | PWWP domain-containing protein | -0.031 | 0.957 |
| A0A4X1UJ47 | Pyridoxal 5'-phosphate synthase | 0.261 | 0.067 |
| A0A5G2QSU5 | Pyruvate dehydrogenase E1 component subunit beta | 0.093 | 0.674 |
| A0A0B8S031 | Pyruvate kinase | -0.450 | 0.072 |
| F6Q2A5 | Quinone oxidoreductase | 0.194 | 0.296 |
| I3L893 | Rab GDP dissociation inhibitor | -0.092 | 0.888 |
| A0A5G2QSK4 | Rab GDP dissociation inhibitor | -0.046 | 0.474 |
| F1RT87 | RAB2A, member RAS oncogene family | 0.227 | 0.078 |
| F1SV14 | Radixin | 0.070 | 0.568 |
| A0A4X1TE59 | Ras-related protein Rab-11A | -0.076 | 0.453 |
| A0A5G2QNH8 | Ras-related protein Rab-21 | -0.055 | 0.923 |
| A0A286ZSH0 | RCSD domain containing 1 | -0.209 | 0.652 |
| **Accession Number** | **Description** | **A vs. D**  **Log2 Fold Difference** | **A vs. D**  **Adjusted**  **P-value** |
| Q6IM77 | Reticulon | 0.354 | 0.145 |
| A0A480LRK1 | Ribonuclease inhibitor | 0.074 | 0.837 |
| A0A4X1UBF3 | RNA helicase | 0.115 | 0.300 |
| A0A4X1WBH3 | RRM domain-containing protein | -0.186 | 0.170 |
| P16960 | Ryanodine receptor 1 | 0.200 | 0.300 |
| A0A5G2QES7 | S-(hydroxymethyl)glutathione dehydrogenase | -0.111 | 0.738 |
| A0A5G2RC35 | S-phase kinase-associated protein 1 | 0.016 | 0.955 |
| A0A480ZCU6 | Sepiapterin reductase | -0.125 | 0.102 |
| A0A287BB72 | Serine/threonine-protein phosphatase 2A 65 kDa regulatory subunit A alpha isoform | 0.168 | 0.518 |
| A0A4X1T2B5 | Serine/threonine-protein phosphatase | -0.049 | 0.924 |
| A0A5G2QNW1 | SERPINE1 mRNA binding protein 1 | 0.042 | 0.905 |
| A0A287BJI6 | Seryl-tRNA synthetase | 0.069 | 0.858 |
| A0A4X1TIY7 | SHSP domain-containing protein | 0.193 | 0.060 |
| A0A4X1VBX8 | Small muscular protein | -0.224 | 0.193 |
| A0A5G2QJK4 | Small ubiquitin-related modifier | -0.233 | 0.300 |
| F1RGN8 | Smoothelin like 2 | -0.270 | 0.375 |
| A0A480ZS84 | Smoothelin-like protein 1 isoform X1 | -0.570 | 0.091 |
| A0A481BDK8 | Striated muscle preferentially expressed protein kinase isoform 1 | 0.120 | 0.480 |
| F1RK10 | Succinate--CoA ligase [ADP-forming] subunit beta, mitochondrial | 0.334 | 0.606 |
| Q29551 | Succinyl-CoA:3-ketoacid coenzyme A transferase 1, mitochondrial | 0.059 | 0.856 |
| A0A287BPT5 | Sulfurtransferase | -0.140 | 0.296 |
| P04178 | Superoxide dismutase [Cu-Zn] | -0.129 | 0.297 |
| A0A480SCG7 | Synaptopodin 2-like protein isoform X1 | 0.024 | 0.857 |
| A0A286ZLL9 | Synaptopodin | -0.129 | 0.606 |
| A0A5G2QN54 | T-complex protein 1 subunit delta | 0.252 | 0.062 |
| A0A4X1UMM6 | Thiamine pyrophosphokinase | -0.126 | 0.550 |
| A0A4X1WA21 | Thioredoxin domain-containing protein | 0.045 | 0.835 |
| A0A287BP50 | Thioredoxin like 1 | -0.185 | 0.217 |
| P82460 | Thioredoxin | -0.047 | 0.630 |
| A0A286ZT96 | TIP41-like protein | -0.008 | 0.986 |
| A0A287ADN4 | Titin-cap | -0.050 | 0.835 |
| F1SRB9 | TPR_REGION domain-containing protein | 0.072 | 0.453 |
| A0A5G2R5E5 | Trans-1,2-dihydrobenzene-1,2-diol dehydrogenase | 0.113 | 0.632 |
| A0A287BQ72 | Transaldolase | 0.040 | 0.893 |
| A0A4X1SX44 | Transgelin | -0.169 | 0.568 |
| A0A287B4E6 | Translationally-controlled tumor protein | -0.250 | 0.294 |
| **Accession Number** | **Description** | **A vs. D**  **Log2 Fold Difference** | **A vs. D**  **Adjusted**  **P-value** |
| A0A5G2R7W5 | Tripeptidyl-peptidase 2 | -0.255 | 0.163 |
| A0A4X1UU78 | tRNA-binding domain-containing protein | 0.263 | 0.568 |
| A0A287BHM1 | Tropomyosin alpha-1 chain | -0.127 | 0.163 |
| A0A4X1VZV4 | Tropomyosin alpha-3 chain | -0.334 | 0.270 |
| A0A4X1SXX4 | Tubulin-specific chaperone A | -0.170 | 0.205 |
| A0A4X1ULI0 | UBC core domain-containing protein | -0.522 | 0.354 |
| A0A5G2RDX3 | UBC core domain-containing protein | -0.188 | 0.098 |
| A0A4X1W9F5 | UBC core domain-containing protein | 0.123 | 0.792 |
| A0A5G2RGI4 | UBC core domain-containing protein | -0.004 | 0.976 |
| A0A5G2QGD9 | Ubiquinone biosynthesis protein | 0.125 | 0.868 |
| F1RHF0 | Ubiquitin carboxyl-terminal hydrolase | 0.395 | 0.100 |
| I3L945 | Ubiquitin carboxyl-terminal hydrolase | -0.241 | 0.400 |
| A0A0B8RZ10 | Ubiquitin-activating enzyme E1 | -0.087 | 0.120 |
| Q06AA9 | Ubiquitin-conjugating enzyme E2 D2 | -0.019 | 0.938 |
| A0A4X1VUF2 | Ubiquitin-like domain-containing protein | 0.060 | 0.913 |
| A0A4X1UYL8 | Uncharacterized protein | 0.077 | 0.606 |
| A0A286ZVQ0 | Uncharacterized protein | 0.084 | 0.792 |
| A0A4X1UAJ6 | Uncharacterized protein | -0.204 | 0.078 |
| A0A4X1ST02 | Uncharacterized protein | 0.123 | 0.607 |
| A0A286ZS27 | Uncharacterized protein | 0.028 | 0.894 |
| A0A4X1W2N4 | Uncharacterized protein | -0.522 | 0.464 |
| F1SEV8 | Uncharacterized protein | -0.058 | 0.709 |
| A0A4X1TLK3 | Uncharacterized protein | 0.482 | 0.354 |
| A0A4X1VCF5 | Uncharacterized protein | 0.012 | 0.957 |
| A0A4X1VES0 | Uncharacterized protein | -0.044 | 0.382 |
| A0A4X1V5J3 | Uncharacterized protein | 0.017 | 0.955 |
| A0A4X1W9X7 | Uncharacterized protein | -0.013 | 0.957 |
| A0A4X1TUT8 | Uncharacterized protein | -0.135 | 0.185 |
| A0A4X1SEG0 | Uncharacterized protein | 0.065 | 0.765 |
| F1RKG8 | Uncharacterized protein | -0.105 | 0.174 |
| A0A4X1UQV2 | Uncharacterized protein | -0.074 | 0.704 |
| A0A4X1TWD6 | Uncharacterized protein | -0.121 | 0.334 |
| A0A5G2Q9D0 | Uncharacterized protein | -0.147 | 0.641 |
| A0A5G2RDF7 | Uncharacterized protein | 0.096 | 0.811 |
| A0A287BKA5 | Uncharacterized protein | -0.227 | 0.595 |
| A0A4X1TZA2 | Uncharacterized protein | -0.555 | 0.165 |
| A0A4X1ST20 | Uncharacterized protein | 0.003 | 0.988 |
| **Accession Number** | **Description** | **A vs. D**  **Log2 Fold Difference** | **A vs. D**  **Adjusted**  **P-value** |
| A0A4X1UNJ7 | Uncharacterized protein | -0.126 | 0.375 |
| A0A4X1T2P8 | Uncharacterized protein | 0.326 | 0.365 |
| A0A4X1VX90 | Uncharacterized protein | -0.180 | 0.126 |
| A0A4X1TU60 | Uncharacterized protein | -0.117 | 0.800 |
| A0A4X1T530 | Uncharacterized protein | 0.112 | 0.856 |
| A0A4X1VUZ8 | Uncharacterized protein | 0.150 | 0.221 |
| A0A4X1W4H6 | Uncharacterized protein | 0.029 | 0.906 |
| A0A4X1T5G0 | Uncharacterized protein | -0.086 | 0.955 |
| A0A4X1VXU3 | Uncharacterized protein | -0.098 | 0.345 |
| A0A4X1VVL9 | Uncharacterized protein | -0.274 | 0.517 |
| A0A4X1T2U8 | Uncharacterized protein | -0.180 | 0.595 |
| A0A4X1UTR8 | Uncharacterized protein | -0.087 | 0.521 |
| A0A4X1W8Z7 | Uncharacterized protein | -0.327 | 0.676 |
| A0A4X1UJP9 | Uncharacterized protein | 0.049 | 0.833 |
| A0A4X1T308 | Uncharacterized protein | 0.017 | 0.937 |
| A0A4X1SPR8 | Uncharacterized protein | 0.033 | 0.955 |
| A0A4X1UCV2 | Uncharacterized protein | -0.015 | 0.965 |
| A0A287A1M4 | Uncharacterized protein | -0.034 | 0.835 |
| A0A287ALC1 | Uncharacterized protein | -0.136 | 0.490 |
| A0A4X1WAZ4 | UTP--glucose-1-phosphate uridylyltransferase | -0.124 | 0.065 |
| A0A4X1VCP8 | UV excision repair protein RAD23 | -0.050 | 0.532 |
| A0A287BBB5 | VAMP associated protein A | -0.051 | 0.695 |
| A0A4X1US63 | Vesicle-associated membrane protein-associated protein B | -0.151 | 0.126 |
| A0A287B073 | Voltage-dependent L-type calcium channel subunit alpha | 0.220 | 0.087 |
| A0A4X1SKZ9 | VWFA domain-containing protein | -0.007 | 0.992 |
| A0A4X1T4Y7 | WH1 domain-containing protein | 0.104 | 0.580 |

^1^Categories A and D were classified by instrumental star probe tenderness. Category A (n = 23): $\bar{x}$ =4.23 kg*,* 3.43-4.55 kg; Category D (n =24): $\bar{x}$ = 6.21 kg, 5.70-7.41.
